# Profiling the Human Phosphoproteome to Estimate the True Extent of Protein Phosphorylation

**DOI:** 10.1021/acs.jproteome.2c00131

**Published:** 2022-05-09

**Authors:** Anton Kalyuzhnyy, Patrick A. Eyers, Claire E. Eyers, Emily Bowler-Barnett, Maria J. Martin, Zhi Sun, Eric W. Deutsch, Andrew R. Jones

**Affiliations:** † Department of Biochemistry and Systems Biology, Institute of Systems, Molecular and Integrative Biology, University of Liverpool, Liverpool L69 7BE, U.K.; ‡ Computational Biology Facility, Department of Biochemistry and Systems Biology, Institute of Systems, Molecular and Integrative Biology, University of Liverpool, Liverpool L69 7BE, U.K.; § Centre for Proteome Research, Department of Biochemistry and Systems Biology, Institute of Systems, Molecular and Integrative Biology, University of Liverpool, Liverpool L69 7BE, U.K.; ∥ European Molecular Biology Laboratory, European Bioinformatics Institute (EMBL-EBI), Cambridge CB10 1SD, U.K.; ⊥ Institute for Systems Biology, Seattle, Washington 98109, United States

**Keywords:** proteomics, database, phosphoproteomics, mass spectrometry, phosphorylation, phosphopeptides, phosphosites, PhosphoSitePlus, PeptideAtlas, proteome, false discovery rate, evolutionary conservation, UniProt

## Abstract

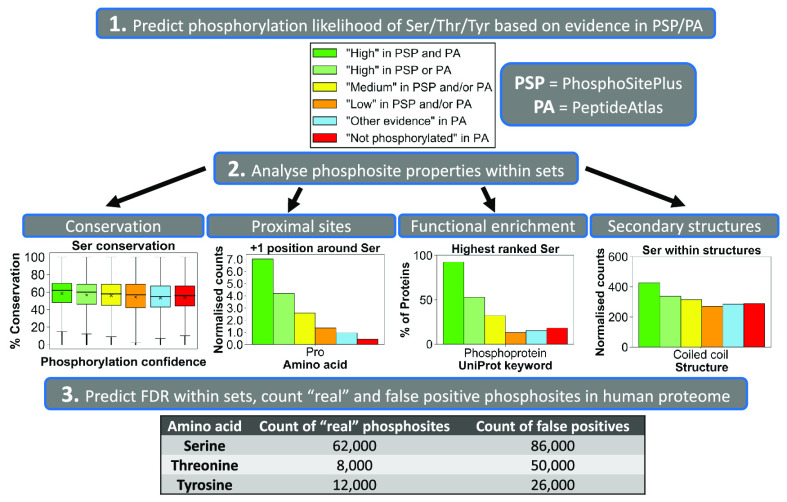

Public phosphorylation databases such as PhosphoSitePlus (PSP) and PeptideAtlas (PA) compile results from published papers or openly available mass spectrometry (MS) data. However, there is no database-level control for false discovery of sites, likely leading to the overestimation of true phosphosites. By profiling the human phosphoproteome, we estimate the false discovery rate (FDR) of phosphosites and predict a more realistic count of true identifications. We rank sites into phosphorylation likelihood sets and analyze them in terms of conservation across 100 species, sequence properties, and functional annotations. We demonstrate significant differences between the sets and develop a method for independent phosphosite FDR estimation. Remarkably, we report estimated FDRs of 84, 98, and 82% within sets of phosphoserine (pSer), phosphothreonine (pThr), and phosphotyrosine (pTyr) sites, respectively, that are supported by only a single piece of identification evidence—the majority of sites in PSP. We estimate that around 62 000 Ser, 8000 Thr, and 12 000 Tyr phosphosites in the human proteome are likely to be true, which is lower than most published estimates. Furthermore, our analysis estimates that 86 000 Ser, 50 000 Thr, and 26 000 Tyr phosphosites are likely false-positive identifications, highlighting the significant potential of false-positive data to be present in phosphorylation databases.

## Introduction

Protein phosphorylation is a fundamental post-translation modification (PTM) that regulates protein function and is well studied in relation to cell signaling pathways and disease.^1−3^ Huge numbers of phosphorylated peptides and sites have been reported and characterized after isolation from human cells using approaches allied to tandem mass spectrometry (LC-MS/MS), focussing primarily on the phosphorylation of “canonical” (established) serine (Ser), threonine (Thr), and tyrosine (Tyr) residues.^4−8^ However, large numbers of “noncanonical” phosphorylation sites have also been annotated on proteins from a variety of sources including human cells.^9^ This additional complexity highlights the ongoing requirement for careful, evidence-based phosphosite identification from mass spectrometric datasets.

Historically, the focused analysis of phosphorylation sites in proteins tended to rely on biochemical analysis including, for example, chromatography and solid-state Edman sequencing.^10−12^ However, while giving confidence in phosphosite identification, such low-throughput approaches are now rare, lacking the depth of coverage needed for most large-scale studies. The dominance of MS approaches has led to the development of multiple strategies to both understand and help mitigate the high levels of phosphopeptide false discovery rate (FDR), particularly in sets of mapped peptide spectral matches (PSMs) that result from LC-MS/MS and sequence database analysis.^13−15^ The goal of such approaches is to separate true identifications from false ones. Even without considering noncanonical phosphorylation (which is likely to be absent in typical phosphoproteomics pipelines due to its acid-labile nature), many confidently identified phosphopeptides possess multiple Ser, Thr, or Tyr residues that could be differentially modified in a given proteolytically generated peptide.^9^ Phosphosite occupancy is variable on any given protein under different biological conditions such that analysis of a peptide containing, for example, two Ser residues that have the potential to be phosphorylated with different dynamics could present evidence for either only one or both being modified, depending on the sample studied.^6,7,16,17^ Many phosphorylation events are also substoichiometric, possibly falling below the limit of detection of certain analyses.^7,16^ As such, careful data handling and statistical processes should be applied, either within the search engine used for peptide mapping or in a downstream software application to calculate additional statistics, such as a local false localization rate (FLR) or conversely the probability that a given site within a peptide is correct or incorrect. Software/algorithms include phosphoRS,^18^ Ascore,^19^ Andromeda’s PTM Score,^20^ and recently released PTMProphet.^21^ We have previously benchmarked the performance of some instrumental parameters and software pipelines for phosphoproteomics,^22^ demonstrating that there is considerable variability in how such scores map to robust statistics, such as local or global FLR, depending on the instrument fragmentation mode and resolution.

Following confident identification of phosphopeptides and localization of given sites, data tend to be compiled from within a single study or across multiple studies (meta-analysis) to determine the extent of evidence for a given site from multiple PSMs. In general, where there are independent observations of PSMs supporting a phosphosite, it can be reasonably assumed that the evidence for a site to be real increases, although to our knowledge, there are no current statistical models to calculate this phenomenon accurately. Multiple PSMs can be observed per identified phosphosite as a result of either different peptide sequences containing that site, or the same peptide sequence being detected several times.^23^ There are some caveats to this logic though, as it is possible for the same PSM to be wrongly assigned to a phosphopeptide multiple times. This can occur if the correct interpretation for the spectrum had a very similar peptide sequence and identical mass to the wrongly assigned phosphopeptide.^16,24^ Although LC-MS/MS and computational analysis is generally recognized as very effective and reliable for phosphosite detection, from each study, it is likely that there is some element of remaining false discovery of peptides and sites wrongly localized, depending on the applied statistical thresholds. This is particularly problematic for studies that set relatively weak thresholds for phosphosite localization (e.g., equating to site probability >0.75) to maximize sensitivity—more true positives may be identified, but at the expense of very large numbers of false positives passing the threshold. A multicenter benchmarking study highlighted some of the challenges in practice, showing considerable variability in the number of true-positive, false-positive, and false-negative sites reported across different laboratories, with particular issues arising when a peptide carried multiple phosphate groups.^25^ Methods and guidelines for FLR are still evolving and not consistently applied in phosphoproteome studies, and so it is likely that most published studies contain considerable numbers of falsely localized phosphosites.^25−29^ This can lead to overestimation of the total number of known true human phosphosites if database providers do not control for FDR across multiple datasets.^30^

One such database is PhosphoSitePlus (PSP) which represents a comprehensive, manually curated, and well-cited resource containing experimentally defined PTMs primarily focusing on phosphorylation.^29^ As of March 2020, PSP encompassed phosphosite evidence across 17 830 human protein sequences, which are defined as canonical in UniProt (representing the most prevalent protein product per gene,^31^ for example). The evidence for phosphorylation comes from manually curated reviews of literature primarily describing tandem MS studies and also low-throughput experiments, or from in-house MS studies.^29^ Interestingly, the majority of phosphosites in PSP only have a single piece of evidence associated with their identification (i.e., there is only one study identifying the phosphosite). As mentioned in the PSP documentation itself, researchers should be cautious when accepting such sites as true positives.^29^ It is possible that many users of PSP are not aware of the need for caution when reviewing or reusing data, and we are not familiar with any previous effort to assess phosphosite FDR within PSP. A second curated proteomics resource is PeptideAtlas (PA)^26^ which is a repository of tandem MS datasets that have been processed through Trans-Proteomic Pipeline to ensure high and consistent quality of phosphopeptide identifications.^32^ The latest PA builds incorporate the use of the PTMProphet algorithm for phosphosite localization where each potential phosphosite within an observed PSM is assigned a probability score between 0 and 1 of being phosphorylated.^21^ As with PSP, researchers should also be careful when accepting sites in PA with only a single piece of identification evidence (i.e., a single associated PSM) as positively identified phosphosites. Instead, phosphosites that not only have multiple PSM observations in PA but also have high phosphorylation probability scores assigned within the majority of those PSMs are most likely to be true-positive identifications. In addition to PSP and PA, other databases containing data on human phosphosites include UniProt, which collates mostly manually curated phosphosites from the literature but is planned to start incorporating high-throughput derived data in later releases;^31^ dbPTM, a server importing data from other resources, but currently unavailable as of July 2021;^33^ and PhosphoDB containing results from a set of studies on phosphopeptides derived from multiple proteases.^34^ Even with easy access to these accumulated phosphorylation site resources, to our knowledge, no estimates have been made to predict the scale of phosphosite FDR across large datasets.

In this work, by profiling the reported human phosphoproteome, we aimed to estimate the false discovery rate of phosphosites with evidence in PSP and/or PA and use these estimates to predict the count of true phosphosites within the currently explored human phosphoproteome. We categorized the sites into sets of various predicted phosphorylation likelihood based on the amount of positive identification evidence reported in PSP and PA, properties not readily available in other databases. Using orthogonal features of phosphosites assigned to these sets, such as evolutionary conservation, sequence properties, and functional annotations, we aimed to demonstrate significant differences between the sets and develop an improved method for independent FDR estimation, which can be used to indicate the extent of true phosphosites within the human phosphoproteome.

## Methods

### Processing and Categorizing Phosphorylation Data in PSP and PA

Phosphorylation data in PeptideAtlas (PA) (2020 build)^26^ was filtered to only include human Ser/Thr/Tyr sites from canonical UniProt protein sequences with at least one PSM observation (1 069 709 sites across 63 616 sequences) (Table S1). The sites were categorized according to the number of PSM observations with a certain phosphorylation probability score assigned by PTMProphet.^21^ The counts of observations with a probability of >0.95 were used as “positive” evidence for site phosphorylation. The counts at a probability threshold of ≤0.19 were used as “negative” evidence in favor of a site being a nonphosphosite. The total number of PSM observations per site was considered to distinguish sites for which ≥10% of all associated PSMs had a PTM probability >0.95, from sites where a small minority (<10%) of associated PSMs had this probability. Based on this, selected confidence categories were applied to predict site phosphorylation likelihood in PA (“*High*”: ≥5 positive observations which accounted for ≥10% of total observations across all probabilities; “*Medium*”: ≥5 positive observations, which accounted for <10% of total observations or 2–4 positive observations; “*Low*”: 1 positive observation; “*Not phosphorylated*”: 0 positive observations and ≥5 negative observations; “*Other*”: site did not fall into any described categories). PhosphoSitePlus (PSP) data (11/03/20 build; Phosphorylation_site_dataset.gz)^29^ was filtered to only include human Ser/Thr/Tyr sites from canonical protein sequences labeled by UniProt identifiers (231 607 sites across 17 830 sequences) (Table S2). The sites were ranked based on the number of times they have been characterized in low/high-throughput studies. The sum of observations across all studies was used to predict site phosphorylation likelihood in PSP (“*High*”: ≥5 observations; “*Medium*”: 2–4 observations; “*Low*”: 1 observation).

### Evolutionary Conservation Analysis

To determine the cross-species conservation of all Ser, Thr, and Tyr sites in the reference human proteome,^31^ which have phosphorylation evidence in PSP and PA, human reference proteome (20 605 sequences, UniProt ID: UP000005640) and the proteomes of 100 eukaryotic species (50 mammals, 12 birds, 5 fish, 4 reptiles, 2 amphibians, 11 insects, 4 fungi, 7 plants, and 5 protists; Table S3) were downloaded from UniProt (UniProt release 2019_10). Each sequence in the human proteome was used as a query in a BLASTp search (BLAST+ 2.10.0 version)^35^ against all 100 eukaryotic proteomes. The BLAST output was processed to extract a top matching significant orthologue (*E*-value of ≤0.00001) from each species for each human target. Human targets were then aligned with their matched orthologues using the MUSCLE algorithm (version 3.8.31)^36^ with default settings if all sequences to be aligned were <2000 amino acids long. If any sequences to be aligned (either the human sequence or any of the orthologue sequences) were ≥2000 amino acids long, two iterations of the algorithm were run using settings for large alignments (-maxiters 2 option).^36^ From the alignments, percentage conservation scores were calculated for every Ser, Thr, and Tyr site within each human target out of 100 (all eukaryotic proteomes) and out of the number of aligned orthologues. Conservation percentages were calculated considering any Ser/Thr substitutions in orthologues, whereby an orthologue was included in the count if, for example, a Thr in its sequence was aligned with a Ser in the target human sequence and vice versa. Conservation data was then cross-referenced with PSP/PA datasets to identify sites in the human proteome with phosphorylation evidence in PSP/PA and determine their conservation. To ensure consistency in terms of proteins and sites used, any human protein target for which it was not possible to calculate site conservation either due to the protein having no matches in BLAST (14 proteins), no significant matches in BLAST (236 proteins), no Ser/Thr/Tyr sites in its sequence (1 protein) or due to failed alignments (10 proteins), was excluded from any further analysis (Table S4). Any human targets labeled with the same UniProt identifier in the reference human proteome, PSP and PA, but which corresponded to different protein sequences across the datasets (73 proteins; Table S4) were also excluded. Conservation was assessed for the remaining targets (Table S5) by linear regression models with nonassumed intercept for a simpler interpretation of slope between phosphosites and nonphosphosites. The average conservation of likely phosphosites (sites ranked “*High*” or “*Medium*” in PSP and/or PA) was plotted against the average conservation of likely nonphosphosites (sites in “*Not phosphorylated*” and “*Other*” sets) within each target protein that had at least three likely phosphosites and three likely nonphosphosites. Conservation scores (%) were also compared across all sites within phosphorylation likelihood sets using box plots.

### Analysis of Amino Acids Adjacent to Phosphosites

Target protein sequences (20 271 sequences; Table S6) were processed to identify amino acids at the −1 and +1 proximal positions adjacent to every Ser, Thr, and Tyr site. If a target sequence ended with a Ser, Thr, or Tyr site then its +1 amino acid was marked as “*Not found*”. For each amino acid, its frequency at each proximal position was first normalized to 1000 and then to its frequency in the prefiltered human reference proteome (expected distribution). Proximal amino acid frequencies around target Ser, Thr, and Tyr in “*High in PSP and PA*” set were compared to those in the “*Not phosphorylated*” set and to the expected amino acid distribution. The comparisons were assessed by Fisher’s exact statistical test^37^ performed using scipy module in Python^38^ with Bonferroni corrections to generate adjusted *p* values. For each amino acid, any significant difference (Bonferroni corrected *p* value <0.001) between the compared sets was used to estimate phosphosite false discovery rate across all phosphorylation likelihood sets. FDR estimates assumed that all sites in the highest phosphorylation likelihood set “*High in PSP and PA*” set were true-positive phosphosite identifications, whereas all sites with the weakest phosphorylation confidence (either the “*Not phosphorylated*” or the “*Other*” set) were nonphosphosites. Therefore, the observed count of a certain proximal amino acid in the “*High in PSP and PA*” (nPos) corresponded to its expected count at 0% FDR, whereas its observed count in the “*Not phosphorylated*” or “*Other*” set (*n*Neg) corresponded to its expected count at 100% FDR. To estimate % FDR in any other phosphorylation likelihood set based on the observed count of the compared proximal amino acid in that set (*n*Obs), we used the following equation
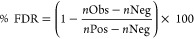


The equation has the effect of estimating what proportion of the observed count (*n*Obs) is explained by assumed false positives (*n*Neg) and what proportion by true positives (*n*Pos). For example, if amino acid X was found at +1 position next to 500 Ser sites in the highest phosphorylation confidence set (0% FDR set; *n*Pos = 500) compared to 10 Ser sites in the “*Not phosphorylated*” set (100% FDR set; *n*Neg = 10), and next to 350 sites in the set of interest (*n*Obs = 350), then pSer FDR within the set of interest would be 31%. This would suggest that 31% of sites in that set behave like false-positive pSer in terms of X amino acid frequency at +1 position, whereas 69% of those sites behave like sites in the highest phosphorylation likelihood set (true pSer).

An average FDR with 95% confidence intervals (CI) was calculated per each likelihood set using all significantly enriched amino acids around a particular target phosphosite and which had an enrichment of >1.5 relative to the expected distribution. Final FDR estimates were used to derive the total number of true-positive (TP) phosphosite identifications across phosphorylation likelihood sets.

To compare FDR/TP estimates between individual PSP and PA sets, the method was replicated using alternative phosphorylation likelihood sets, where sites were categorized according to the highest amount of positive phosphorylation evidence from one database (at least one observation at PTM probability >0.95 in PA; at least one observation in PSP), without taking into account any evidence in the other. Phosphosite FDR estimates within “*High*” sets in each database were presented as a weighted average between FDR estimates in sites ranked “*High*” in that database only and sites ranked “*High*” in both PSP and PA. For example, the FDR in “*High in PA*” set was a weighted average of FDR estimates in “*High in both*” set and “*High in PA only*” set.

To analyze phosphosites in UniProt, phosphorylation data for the reference human proteome was downloaded directly from UniProt (June 2021 version; release 2021_04) and processed to split phosphosites according to associated evidence codes from the Evidence and Conclusion Ontology (ECO:0007744—combinatorial computational and experimental evidence imported from large-scale experiments; ECO:0000269—manually annotated experimental evidence; ECO:0000250—similarity evidence based on orthologous sequence). Any target proteins removed earlier (Table S4) were also removed from this analysis. The resulting protein sequences (*n* = 9481) and sets of Ser, Thr, and Tyr phosphosites were analyzed in terms of adjacent amino acids and conservation using the above method. Phosphosite FDR was calculated for the large-scale study set (ECO:0007744) using “*High in PSP and PA*” as 0% FDR set and “*Not phosphorylated*” as the 100% set.

### Functional Enrichment Analysis

All protein sequences in the filtered reference human proteome (Table S6) were categorized into sets according to what their highest-ranked Ser, Thr, and Tyr site was in terms of phosphorylation evidence (“*High in PSP and PA*”, “*High in PSP or PA*”, “*Medium in PSP and/or PA*”, “*Low in PSP and/or PA*”, “*Other in PA*”, “*Not phosphorylated”* and “*No evidence in PSP or PA*”). Each protein set within Ser, Thr, and Tyr datasets was analyzed with DAVID (version 6.8)^39^ using all proteins in filtered proteome with any Ser, Thr, or Tyr evidence in PSP or PA (16 296, 14 565, and 12 912 proteins, respectively) as control background. Protein sets containing no reported evidence in PSP or PA were searched against a background of all proteins in the filtered reference proteome to determine any differences in their functional enrichment compared to proteins with PSP/PA evidence. Per each set searched, the top 10 (where possible) significant (Benjamini–Hochberg corrected *p* value <0.05) functional terms with the highest percentage of proteins mapped were identified, replacing any near-synonymous terms with additional terms from outside the initial top 10. All target protein sets were also searched in UniProt (release 2020_04) to determine the percentage of proteins mapped to UniProt keywords “*Phosphoprotein*”, “*Alternative splicing*”, “*Nucleus*”, “*Transcription*”, “*Acetylation*”, “*Membrane*”, “*Glycoprotein*”, “*Signal*”, and “*Disulfide bond*”.

### Secondary Structure Analysis

Categorized Ser, Thr, and Tyr sites in filtered reference human proteome were mapped to protein structures (β strand, helix, turn, and coiled coil) described for those proteins in UniProt (release 2020_04) (Tables S5 and S7). Any target proteins searched in UniProt which were marked as obsolete (15 proteins) or represented different sequences despite being labeled with the same identifier (25 proteins) were removed further and marked as “*NA*” (Table S5). Normalized (to 1000) counts of target amino acids within protein structures were assessed with Fisher’s exact statistical test^37^ using the scipy module in Python^38^ to generate *p* values and indicate any significant enrichment (*p* < 0.05) between “*High in PSP and PA*” set and the “*Not phosphorylated*” set. The method was also applied separately for Ser sites, which had phosphorylation evidence in UniProt, and which were mapped to the described phosphorylation likelihood sets based on PSP/PA evidence.

## Results and Discussion

### Categorizing All Ser, Thr, and Tyr Annotated Phosphosites in the Human Proteome

**Figure 1:**
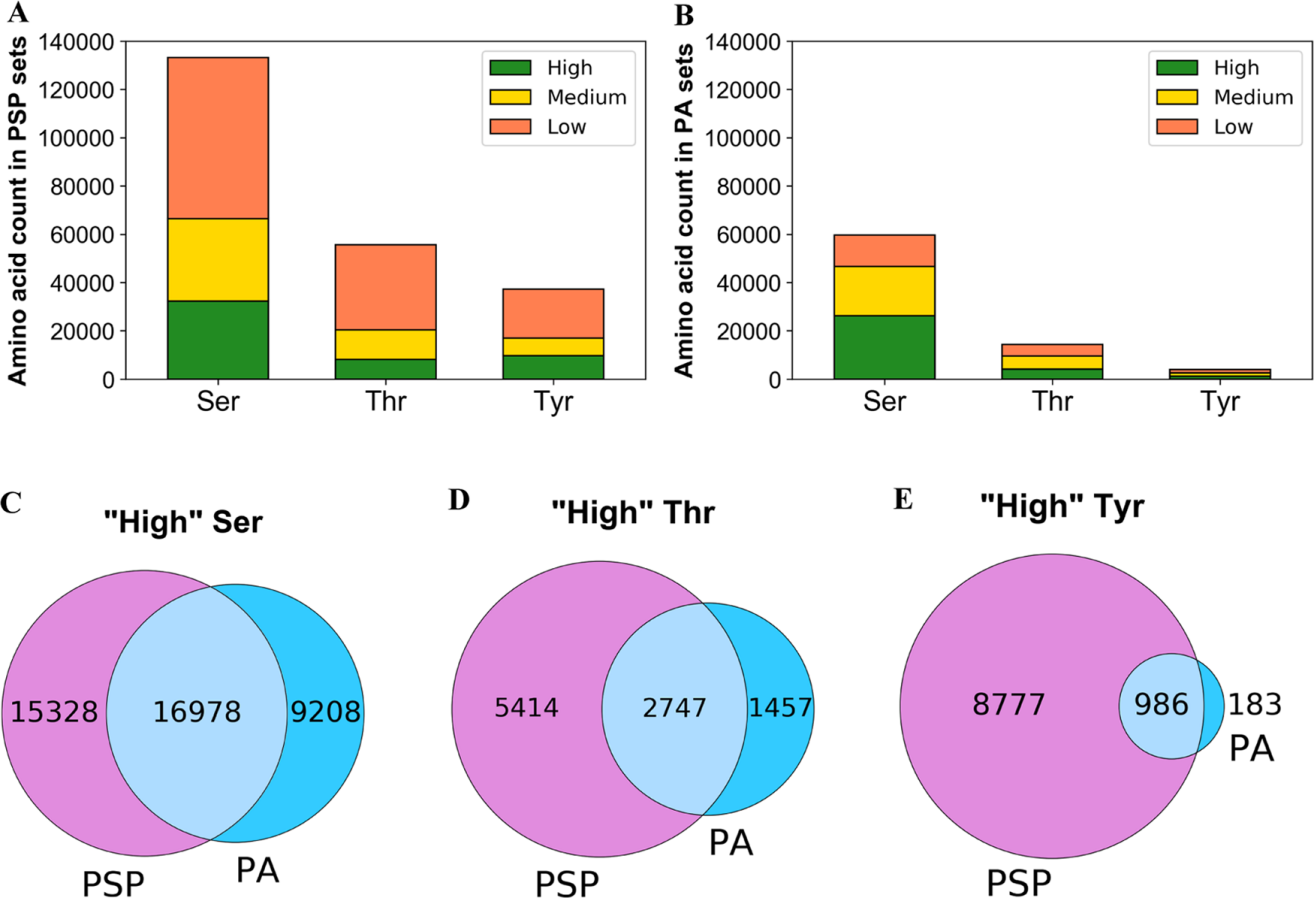
Distribution of serine (Ser), threonine (Thr), and tyrosine (Tyr) phosphosites from UniProt’s reference human proteome that have any positive identification evidence in (A) PhosphoSitePlus (PSP) or (B) PeptideAtlas (PA) based on established phosphorylation likelihood sets (see the Methods section). Venn diagrams provide the counts of (C) Ser, (D) Thr, and (E) Tyr sites ranked “*High*” in PSP (left), PA (right), and both resources (overlap).

We first ranked all Ser, Thr, and Tyr phosphosites in PA and PSP in the filtered reference human proteome according to the amount of accumulated identification evidence (Figure 1 and Tables 1 and S5). The majority of Ser, Thr, and Tyr sites (50.1, 63.3, and 54.3%, respectively) with phosphorylation evidence in PSP were placed into the “*Low*” phosphorylation likelihood set, meaning that there was only a single piece of evidence supporting their positive identification (Figure 1A). Furthermore, out of all analyzed Ser, Thr, and Tyr sites with at least one observation at PTM probability >0.95 in PA (suggesting a positive phosphosite identification), 21.7, 34.0, and 33.5%, respectively, were placed in the “*Low*” set (Figure 1B and Table 1), highlighting that a considerable amount of potential phosphosites only had one piece of positive identification evidence across both databases.

**Table 1:** Categorizing Serine (Ser), Threonine (Thr), and Tyrosine (Tyr) Sites from UniProt’s Reference Human Proteome into Phosphorylation Likelihood Sets Based on Available Phosphorylation Evidence in PhosphoSitePlus (PSP) and PeptideAtlas (PA)

Phosphorylation likelihood set	Phosphorylation evidence per site	Ser count	Thr count	Tyr count
High in PSP	5+ pieces of evidence	32 306	8161	9763
Medium in PSP	2–4 pieces of evidence	34 154	12 197	7228
Low in PSP	1 piece of evidence	66 777	35 173	20 191
High in PA	5+ observations at PTM score >0.95 which is ≥10% of total observations	26 186	4204	1169
Medium in PA	5+ observations at PTM score >0.95 which is <10% of total observations OR 2–4 observations at PTM score >0.95	20 517	5297	1460
Low in PA	1 observation at PTM score >0.95	12 950	4895	1324
Not phosphorylated	0 observations at PTM score >0.19 AND 5+ observations at PTM score ≤0.19 AND no evidence in PSP	13 892	8462	2184
Other sites	At least 1 observation in PA but does not fall into any other PA categories AND no evidence in PSP	60 221	35 000	10 009

Interestingly, we found that in the human proteome there were more Tyr sites assigned to “*High*” set in PSP (5+ observations) than Thr sites (Figure 1A and Table 1), indicating a higher initial proportion of pTyr compared to pThr in PSP. The high prevalence of likely true Tyr phosphosites in the PSP dataset could have been a result of in-house studies which identified large numbers of pTyr sites using immunoaffinity strategies not suitable for pSer/pThr discovery,^40,41^ and studies that have not been officially published.^29^

From PA, it is possible to identify sites for which covering phosphopeptides are observed, but for which the modifications are only localized to other sites in the same peptides, thus providing strong evidence for likely nonphosphosites. Sets of potential Ser, Thr, and Tyr nonphosphosites were therefore established based initially on evidence in PA (Table S8). Those sets were then cross-referenced with data in PSP to determine whether PSP contained any sites ranked as nonphosphosites in PA. Interestingly, we found that 2489 Ser, 1341 Thr, and 891 Tyr sites assigned to the “*Not phosphorylated*” set in PA were found to have evidence in PSP (Table S9). In fact, out of those potential PA nonphosphosites, 146 Ser, 97 Thr, and 293 Tyr sites were placed into “*High*” phosphorylation likelihood set according to PSP evidence (Table S9). This strongly indicated the presence of potential false positives in PSP and/or false negatives in PA. For example, Ser42 in protein P17066 (HSPA6) and Ser59 in Q8N488 (RYBP) had 8 and 6 phosphosite identification references in PSP, respectively (mostly from in-house MS studies), but had no positive identification evidence in PA or any mention in UniProt^31^ (Table S5). On the other hand, Ser4 in P15927 (RPA2) had 33 phosphosite identification references in PSP and was also mentioned in UniProt’s annotations, but has never been positively localized in any of its 127 associated PSMs in PA (Table S5). To eliminate potential false assignments when considering evidence in both PSP and PA, a site was only categorized as a nonphosphosite if it had no evidence in PSP in addition to having “negative” phosphorylation evidence in PA (Table 1). As a result, we established final negative control sets containing 13 892 Ser, 8462 Thr, and 2184 Tyr sites. Similar adjustments were made to the “*Other*” PA set (sites in that set must have no evidence in PSP) which contained the majority of analyzed PA sites (Table 1).

Having further cross-referenced sets of sites of various phosphorylation likelihood between PSP and PA (Table S9), we established a “gold standard” set of phosphosites, all of which had “*High*” phosphorylation likelihood according to both PSP and PA evidence (Table S10). This set contained 16 978 Ser, 2747 Thr, and 986 Tyr highly likely true phosphosites (Figure 1C–E and Table S10). As for the general agreement between PSP and PA in terms of phosphorylation evidence, we found that 37.7% Ser, 20.5% Thr, and 9.10% Tyr sites with PSP evidence also had at least one observation at PTM probability >0.95 in PA (Table S9). This variation in phosphosites observed between the two databases can be explained by the likely use of different methods for phosphosite detection and localization between PA and the sources referenced in PSP, as well as due to a considerable presence of random false positives in both datasets before thresholding has been applied (see the Introduction section).

### Evolutionary Conservation Analysis

**Figure 2:**
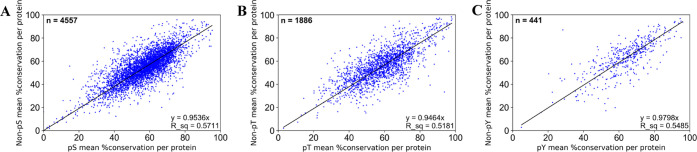
Mean % conservation across 100 eukaryotic species of likely (A) Ser, (B) Thr, and (C) Tyr phosphosites and corresponding likely nonphosphosites within each target protein (*n* = number of proteins analyzed). The regression coefficient (*R*^2^) is given by “R_sq”.

Phosphoproteomes from all species are constantly evolving, although many ancient phosphosites are conserved across species and taxa, increasing the likelihood of them being functionally relevant.^42−44^ In our analysis, we determined the conservation of all potential Ser, Thr, and Tyr phosphosites and nonphosphosites in UniProt’s filtered reference human proteome across 100 eukaryotic species (Table S5), weighed toward vertebrates, but also including examples of insects, plants, and unicellular eukaryotes (Table S3). In our first analysis, we explored the mean conservation of phosphosites and nonphosphosites per protein (at least three of each present per protein) and performed a correlation analysis across all proteins (Figure 2). We fitted linear regression models through the origin, under the theory that proteins unique to humans would have zero conservation for both phosphosites and nonphosphosites. We found great variation between the conservation of both site types, ranging from near zero to 100%, which was mostly dependent on the overall conservation of the protein sequence. However, based on the generated linear regression models, we concluded that on average, Ser, Thr, and Tyr phosphosites (“*High*” or “*Medium*” in PSP and/or PA) were around 4.6, 5.4, and 2.0%, respectively, more conserved across all 100 eukaryotes than corresponding likely nonphosphosites (sites in “*Not phosphorylated*” and “*Other*” sets) within analyzed proteins when allowing Ser/Thr substitutions toward the conservation score (Figure 2). Similar results were obtained when assessing phosphosite conservation only across found orthologues for each protein (Figure S1). The results (Figures 2 and S1) provide additional evidence that phosphosites are generally more conserved than nonphosphosites.^42,43,45^ The difference in conservation is thus subtle and variable, but statistically robust. Furthermore, in our analyzed sets of proteins which had at least three likely phosphosites and three likely nonphosphosites, we found 104, 88, and 19 proteins where conservation of Ser, Thr, and Tyr likely phosphosites, respectively, was at least 20% higher than conservation of likely nonphosphosites (Table S11).

**Figure 3:**
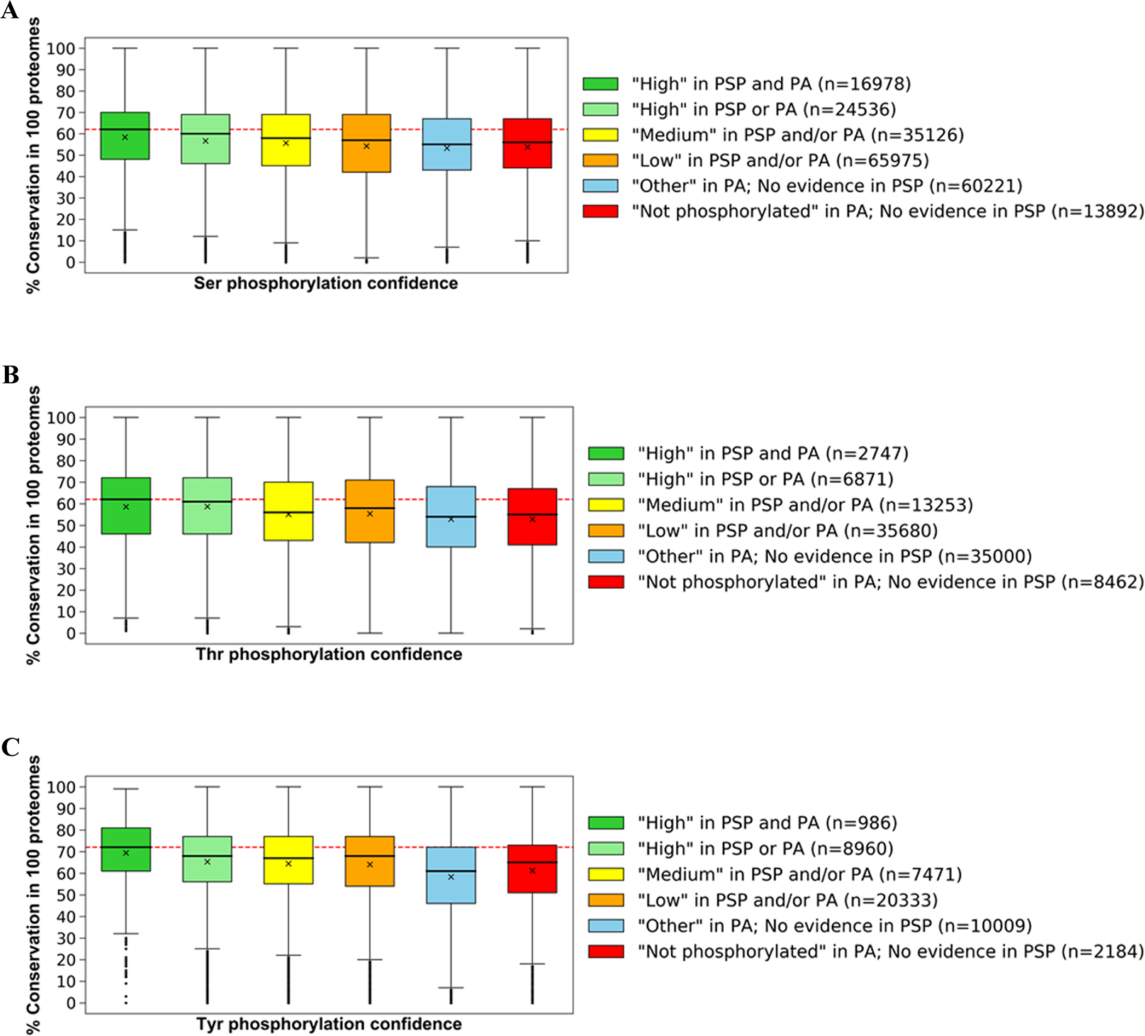
Box plots of conservation percentages (%) across 100 eukaryotic species of human (A) Ser, (B) Thr, and (C) Tyr sites categorized across established phosphorylation confidence sets based on PSP and PA evidence. Within each box, a horizontal line represents median % conservation and (x) symbol represents mean % conservation per group. Each box extends from the 25th to the 75th percentile of each set’s distribution of conservation % values. Vertical lines extending from the boxes correspond to adjacent values. Dots (•) represent outlier values. The red line shows median % conservation in “*High in PSP and PA*” set for visual comparison.

We next compared the conservation of all sites split by phosphorylation likelihood sets (Figure 3) and observed that sites in the highest phosphorylation likelihood set (“*High in both PSP and PA*”) had the highest average conservation across all 100 eukaryotic proteomes considering Ser/Thr substitutions (average conservation of 58.4, 58.6, and 69.4% across 16 978 Ser, 2747 Thr, and 986 Tyr sites, respectively) (Figure 3 and Table S12). In comparison, the sites in “*Low in PSP and/or PA*” set had slightly lower average conservation scores of 54.3, 55.4, and 64.0% in 35 126 Ser, 13 253 Thr, and 7471 Tyr sites, respectively (Figure 3 and Table S12). Assuming that high conservation is a property of true phosphosites, the results (Figure 3) show that this property was observed more frequently in higher phosphorylation likelihood sets compared to lower ones suggesting higher potential phosphosite FDR in sets with less phosphorylation evidence.

There were numerous cases in our analysis of likely nonphosphosites and sites with “*Low*” phosphorylation likelihood where amino acid conservation was also high compared to likely phosphosites, indicative of a conserved function for these amino acids in, for example, catalysis or a biomolecular interaction that is unrelated to phosphorylation. Furthermore, we found 64, 30, and 6 proteins in which the average conservation across 100 eukaryotes of Ser, Thr, and Tyr likely nonphosphosites, respectively, was at least 20% higher than the conservation of corresponding likely phosphosites (Table S11). It is possible that the predicted phosphosites within those proteins were either false positives or were nonfunctional true phosphosites, explaining the comparative weaker selective pressure. In fact, previous reports estimated that as many as 65% of known phosphosites may be nonfunctional as individual sites (although may have a more general structural role) due to limited kinase specificity and therefore have similar evolution rates compared to nonphosphosites which would explain the observed trends.^46,47^ It is also possible that some proteins were formed by recent gene fusion events leading to regions containing phosphorylation sites only found in a few closer related orthologues (low conservation), with other protein domains being more highly conserved. In addition, higher evolutionary rates in closely related species (primates, for example) could lead to new protein functions unique to that group of species, further explaining low conservation of some phosphosites in our analysis.

We further note that Tyr sites in the highest phosphorylation likelihood set (“*High in PSP and PA*”) had a higher mean conservation (69.4%) compared to Ser/Thr sites in that set (58.4 and 58.6%, respectively) (Figure 3 and Table S12). There are several possible explanations for this result, including the idea that pTyr is under stronger conservation pressure (i.e., mutations cannot easily be tolerated) in animals that make up the vast majority (84/100) of the species analyzed (Table S3). It is also possible that there is a degree of experimental bias due to the comparison of the much larger set of pSer/pThr to pTyr. The typically higher data quality for pTyr, enhanced by the availability of epitope-specific monoclonal antibodies may also contribute to this phenomenon.

### Analysis of Amino Acids Adjacent to Phosphosites

Amino acids directly adjacent to known phosphorylation sites are often involved in optimizing substrate capture for subsequent phospho-transfer by the kinase enzymatic machinery.^48−50^ Multiple reports specifically highlight the importance of proline (Pro) in the mechanism of phosphorylation for families of kinases such as the cyclin-dependent kinases, mitogen-activated protein kinases, and, more recently, the centrosomal kinase PLK4.^48,51−56^ Consequently, there is a high prevalence of Pro in numerous phosphorylation motif sequences as part of Ser/Thr-Pro combinations.^57,58^

**Figure 4:**
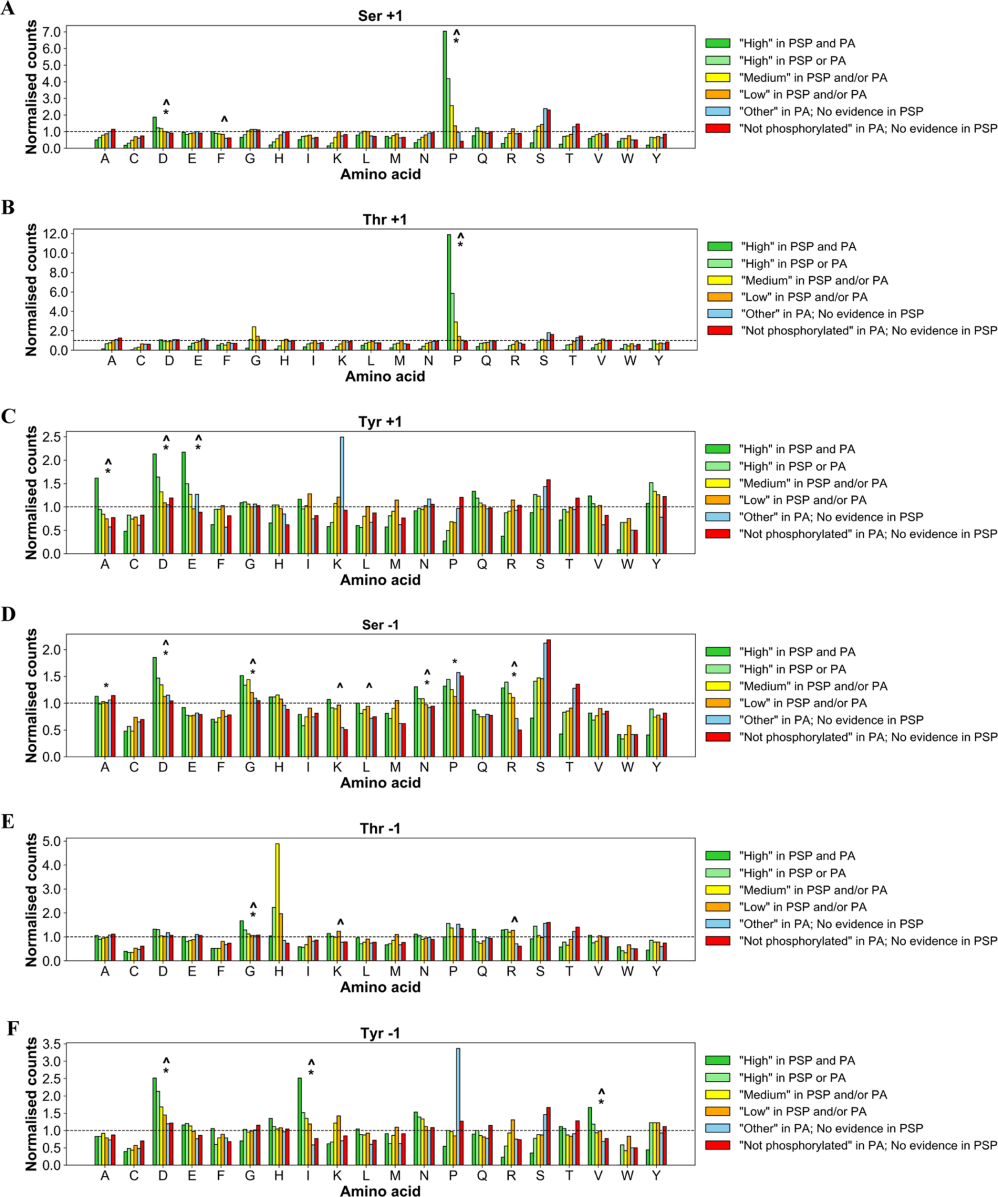
Counts of proximal amino acids positioned at (A) +1 around Ser; (B) +1 around Thr; (C) +1 around Tyr; (D) −1 around Ser; (E) −1 around Thr; and (F) −1 around Tyr sites of various phosphorylation likelihood based on evidence in PSP and PA, normalized to the observed distribution of those amino acids in human proteome (represented by dotted baseline fixed at 1). Significant (Bonferroni corrected *p* value <0.001) enrichment of proximal amino acids in the “*High in PSP and PA*” set is highlighted by the caret symbol (∧) compared with the “*Not phosphorylated*” set, and an asterisk (*) compared to the expected amino acid distribution.

In our analysis, we identified the frequency of −1 and +1 amino acids relative to a possible phosphosite and compared it across different sets of sites ranked by the relative strength of phosphorylation evidence in Table 1. We found a strong enrichment of Pro at the +1 position next to Ser and Thr sites in the reference human proteome that were placed in the set with the most phosphorylation evidence (“*High in PSP and PA*”) (Figure 4A,B and Table S13). In fact, Pro was observed at the +1 position next to 44.3 and 74.9% of all Ser and Thr sites, respectively, in that set (Table S13). The enrichment of Pro at +1 position around those sites was significant (adj. *p* value <0.001) in relation to the normalized distribution of Pro in the human proteome, where it is, in fact, only the sixth most observed amino acid (Table S13). The normalized number of observations of Pro at +1 relative to Ser and Thr sites in the highest phosphorylation likelihood set was also significantly (adj. *p* value <0.001) higher than around Ser/Thr sites in the “*Not phosphorylated*” set (Figure 4A,B), where only 2.68% of Ser and 5.67% of Thr sites had Pro at +1 position (Table S13). Therefore, the enrichment of Pro around highly likely Ser and Thr phosphosites suggests that this feature, among others, can be used as a differentiating characteristic for phosphosites compared to nonphosphosites.

We also found a significant enrichment of Asp at +1 position next to Ser sites in the highest phosphorylation likelihood set (Figure 4A). To explain this, we linked the sequences containing those sites to phosphorylation motifs which commonly feature Ser-Asp combinations, including those phosphorylated by Casein kinase II.^57,59^ At the −1 positions around target Ser, we found significant enrichment (adj. *p* value <0.001) of Asp and Gly in the highest phosphorylation likelihood set compared to “*Not phosphorylated*” set (Figure 4D). It is possible that the observed enrichment was due to the presence of those amino acids within substrate motifs of Casein Kinase II, CDK5, PKC, and MEKK,^57^ suggesting high prevalence of potential true Ser phosphosites. Similar conclusions were made for the enrichment of Gly at −1 around Thr sites in the highest phosphorylation likelihood set (Figure 4E), which was linked to possible Gly–Thr combinations within PKA, ERK1, and ERK2 kinase substrate motifs.^57^

We compared the frequency of significantly enriched amino acids (Bonferroni corrected *p* value <0.001; enrichment >1.5) across the sites within different phosphorylation likelihood sets and used the comparison to estimate phosphosite false discovery rate across those sets. Using the counts of all four enriched amino acids (Asp and Pro at +1; Asp and Gly at −1) around Ser sites of various phosphorylation likelihood (Figure 4A,D) and working under the assumption of FDR = 0% in set 1 “*High in PSP and PA*”, we estimated average Ser phosphosite FDR = 49% (CI ± 12%) in set 2 “*High in PSP or PA*”; FDR = 54% (CI ± 25%) in set 3 “*Medium in PSP and/or PA*”; FDR = 84% (CI ± 11%) in set 4 “*Low in PSP and/or PA*” and FDR = 91% (CI ± 4%) in the “*Other*” set. Similarly, using the enrichment of Pro at +1 and Gly at −1 around target Thr sites, we estimated Thr phosphosite FDR = 59% (CI ± 7%) in set 2; FDR = 86% in set 3 (CI ± 8%); FDR = 98% (CI ± 5%) in set 4 and FDR = 99% (CI ± 1%) in the “*Other*” set (Tables 2A and S14). Our FDR estimates clearly highlight that the majority of Ser and Thr sites with just one piece of phosphosite identification evidence are likely false-positive identifications, and users of these databases can reasonably assume that if a site does not have multiple levels of evidence, then it is unlikely to represent a true phosphorylation site.

**Table 2:**
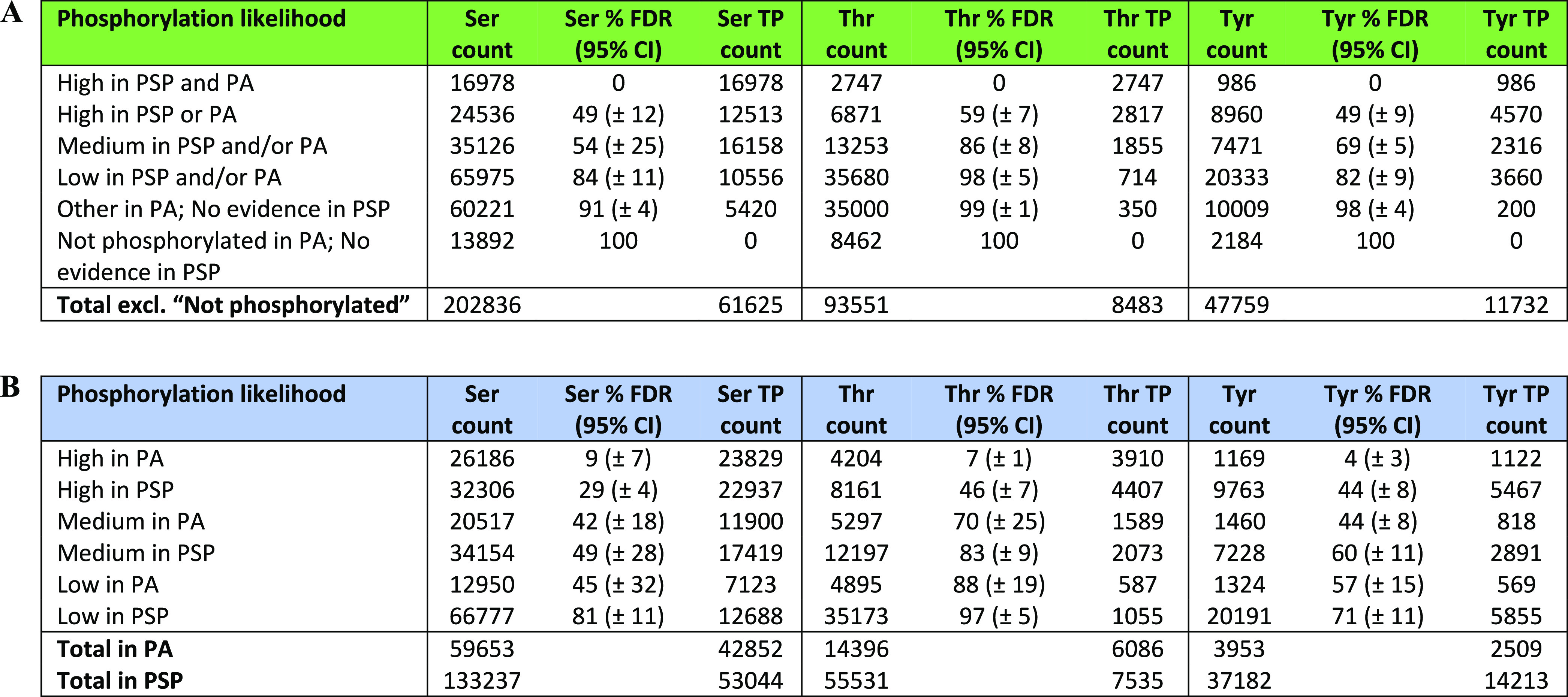
Counts of Estimated True-Positive (TP) Serine (Ser), Threonine (Thr), and Tyrosine (Tyr) Phosphosites within Sets of Various Phosphorylation Likelihood Based on (A) Combined Evidence and (B) Individual Positive Identification Evidence in PhosphoSitePlus (PSP) or PeptideAtlas (PA)^a^

a For each set, TP counts were derived from the FDR estimates within the set and the overall count of target amino acids in the set.

In our analysis of proximal sites around target Tyr, we found a significant enrichment (adj. *p* value <0.001) of Ala, Glu, and Asp at +1 positions, in addition to enriched Ile, Val, and Asp at −1 in “*High in PSP and PA*” set compared to “*Not phosphorylated*” set (Figure 4C,F). We were able to link the enrichment of those proximal sites to their possible involvement in various phosphorylation motifs including EGFR and Abl kinase substrate motifs; PTP1B and PTPRJ phosphatase substrate motifs, and multiple SH2 domain binding motifs,^57,60^ therefore indicating a higher frequency of true Tyr phosphosites in the highest confidence set compared to other sets. Using the frequencies of all six enriched proximal amino acids around target Tyr in “*High in PSP and PA*” (Figure 4C,F), we estimated FDR = 49% (CI ± 9%) in set 2 “*High in PSP or PA*”, FDR = 69% (CI ± 5%) in set 3 “*Medium in PSP and/or PA*”, FDR = 82% (CI ± 9%) in set 4 “*Low in PSP and/or PA*”, and FDR = 98% (CI ± 4%) in the “*Other*” set (Tables 2A and S14).

Our FDR estimates varied depending on the selected enriched proximal amino acid in the highest phosphorylation likelihood set (Table S14), and thus the FDR estimates obtained with our method should be seen as approximate indicators of the extent of false positives in a set of sites with quantifiable phosphorylation evidence.

Based on our Ser, Thr, and Tyr phosphosite FDR estimates, we predicted that there were around 62 000 Ser, 8000 Thr, and 12 000 Tyr true-positive (TP) phosphosite identifications in the human proteome that were supported by evidence in PSP and/or PA (Table 2A). Furthermore, the results suggested that 86 000 Ser, 50 000 Thr, and 26 000 Tyr sites with positive phosphorylation evidence in PSP and/or PA (sites in “*High*”, “*Medium*”, and “*Low*” sets) were false positives (Table 2A). Interestingly, the estimated count of Tyr TPs was higher than the count of Thr TPs, which goes against the general understanding of threonine phosphorylation being more prevalent than tyrosine,^6^ although it is difficult to estimate the underlying true distributions, given experimental biases due to availability of different tools and methods. Our results are influenced because there are initially more Tyr sites with “*High*” or “*Medium*” phosphorylation evidence than Thr sites, particularly in PSP (Figure 1A and Table 2A), where there has been a strong focus to identify Tyr sites using in-house methods. The ratio of the count of sites that have been recorded as “*High*” in both databases is however 16 978 (pSer), 2747 (pThr), and 986 (pTyr), following more closely previously reported estimates of phosphorylation site frequency. It thus remains to be seen if the pTyr sites reported in PSP, but without independent evidence are true or false.

Using the same method, we compared phosphosite FDR between PSP and PA sets by considering positive phosphorylation evidence (“*High*”, “*Medium*”, or “*Low*” sets) in one database without taking into account any evidence in the other (Figure S2 and Table S15). The analysis revealed a generally lower FDR per each set in PA compared to the respective set in PSP, overall suggesting that a higher proportion of analyzed sites in PA are true phosphosites compared to the analyzed sites in PSP (Tables 2B and S15).

As noted in the Introduction, manually curated evidence for phosphorylation sites is also collated in UniProt. However, while this resource provides information pertaining to the publication providing this evidence, the number of individual observations is not reported, preventing a matched analysis being performed with PSP/PA. Nevertheless, we were able to extract all phosphorylation data from the human reference proteome in UniProt and separate phosphosites into sets according to the type of manually curated phosphosite evidence (experimental evidence, combinatorial computational and experimental evidence from large-scale experiments, sequence similarity with an orthologous protein). As before, the sets were analyzed in terms of adjacent amino acids around target phosphosites (Figure S3A–F). Due to potential phosphosite differences and biases associated with different discovery methods (motif frequency, for example), we suggest that our method should only be used to analyze sites from high-throughput studies because it was built primarily using sites of similar evidence type. This is further evident from the conservation analysis of UniProt sites (Figure S3H,I), which revealed different conservation patterns between the set of sites identified by large-scale studies and the other UniProt sets. As a result, we were able to estimate FDR for a set of UniProt sites with evidence from large-scale proteomics studies (Figure S3G). In that set, we estimated average pSer FDR = 7% (CI ± 8%); pThr FDR = 22% (CI ± 14%) and pTyr FDR = 6% (CI ± 7%) (Figure S3G), suggesting that there is a much higher proportion of true-positive phosphosites in UniProt compared to PSP or PA datasets. The FDR difference between pSer and pThr follows the statistical expectation from analyses of large datasets with unbalanced counts of true positives for different residues. For example, if a study reported 1200 phosphosites at 5% FDR, of which 1000 are pSer and 200 are pThr, the false positives (∼60) would assort approximately equally across pSer (∼30 out of 1000 i.e., 3% FDR on pSer) and pThr (∼30 out of 200 i.e., 15% on pThr), meaning that in the vast majority of studies (which do not correct for this issue) the general pThr FDR will be significantly higher than for pSer. For pTyr, the majority of sites comes from separate studies that specifically enrich for pTyr via antibodies, which likely accounts for the pTyr FDR being similar to the pSer FDR.

### Functional Enrichment Analysis

In our analysis, we categorized all 20 271 proteins in the filtered human reference proteome (Table S5) according to what their highest-ranked Ser, Thr, and Tyr site was based on phosphorylation likelihood sets in Table 1. The resulting sets (Table S16) were analyzed in DAVID^39^ to compare functional enrichment patterns between phosphorylation likelihood sets. First, we found that across all datasets (Ser, Thr, and Tyr) the protein sets containing sites ranked “*High in both PSP and PA*” were associated with the most significant (Benjamini–Hochberg adj. *p* value <0.05) functional groups (Figure S4) suggesting their functional coherence i.e., sharing mappings to keywords, ontology terms or pathways. Interestingly, proteins with sites from “*Low in PSP and/or PA*” set as their highest-ranked site and proteins which did not have any evidence phosphorylation evidence (“*No evidence in PSP or PA*” set) were also enriched for numerous functional categories suggesting that they too share some functional properties (Figure S4). Proteins containing sites from the “*Not phosphorylated*” set as their highest-ranked Ser/Thr/Tyr site were enriched for one significant functional group in the case of Tyr dataset and no functional groups in the case of Ser/Thr datasets, which was likely due to small protein sample size in those sets.

**Figure 5:**
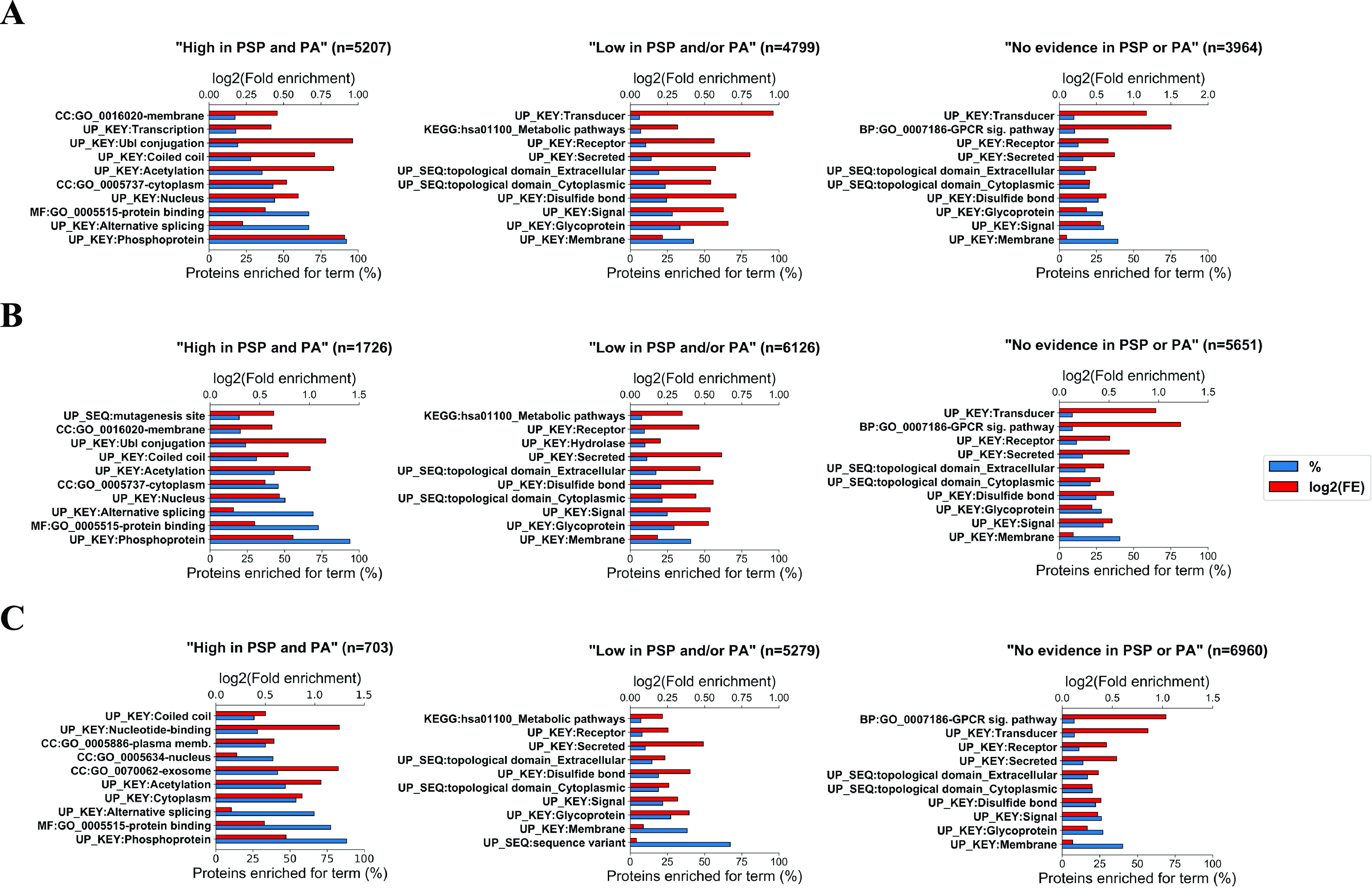
Top 10 functional categories for which protein sets containing various highest-ranked (A) Ser, (B) Thr, and (C) Tyr sites based on the amount of available phosphorylation evidence (“*High in PSP and PA*”, “*Low in PSP and/or PA*”, “*No evidence in PSP or PA*”) were significantly enriched in DAVID (Benjamini–Hochberg corrected *p* value <0.05). For each protein set, the % of proteins enriched for a particular functional category is given as well as the log 2(fold enrichment) for that set. The number of proteins in each set is presented by *n*.

To investigate this further, we compared the top 10 enriched functional groups between the protein sets and found that proteins containing Ser, Thr, and Tyr sites with the most phosphorylation evidence (“*High in PSP and PA*” set) were significantly enriched for categories and terms associated with phosphorylation such as “*Phosphoprotein*”, “*Transcription*”, “*Nucleus*”, and “*Alternative splicing*” (Figure 5), suggesting that those proteins were true phosphoproteins. There is a risk of generating circular evidence here, as the enriched term “*Phosphoprotein*” is a UniProt keyword, and will have been annotated based on literature evidence, potentially shared with PSP. UniProt does not routinely load phosphorylation evidence from high-throughput datasets, and so classifications of phosphoproteins are generally independent of evidence used in PA. Other enriched keywords have also likely been determined based on independent evidence, and thus we believe are unbiased observations of our sets. Overall, 92.3, 93.9, and 88.2% of proteins containing Ser, Thr, and Tyr sites of the highest phosphorylation likelihood, respectively, were enriched for the term “*Phosphoprotein*”, which, as per description in UniProt, is a term assigned to a “*protein which is post-translationally modified by the attachment of either a single phosphate group, or of a complex molecule, such as 5*′*-phospho-DNA, through a phosphate group*”.^31^ Furthermore, those proteins were enriched for “*Acetylation*” (Figure 5) which in some cases might indicate phosphorylation since crosstalk between acetylation and phosphorylation has been frequently reported,^61,62^ alongside other modifications such as O-glycosylation.^63^ Another enriched function is “*Alternative splicing*” (Figure 5) which is known to be controlled by reversible phosphorylation,^64^ further indicating that those proteins likely contain functional phosphosites. However, it is possible that this enrichment could correlate with the depth of analysis of the mentioned proteins rather than their phosphorylation likelihood since extensively studied gene products (and abundant proteins with more easily detectable phosphosites) are likely to have better quality data associated with isoform identification and be consequently linked to “*Alternative splicing*”.

In comparison, proteins that only had sites from “*Low in PSP and/or PA*” set as their highest-ranked Ser, Thr, and Tyr sites (i.e., proteins which did not have sites with strong phosphorylation evidence) were not enriched for clear phosphorylation-associated terms and were instead enriched for categories such as “*Glycoprotein*”, “*Signal*” and “*Disulfide bond*” and “*Membrane*” (Figure 5), suggesting that the majority of those proteins were likely nonphosphoproteins and their associated phosphosites with weak evidence were therefore likely false positives. Assuming that sites with no phosphorylation evidence in PSP or PA are likely nonphosphosites (although it is possible that phosphorylation has not been investigated or localized yet), potential high FDR in the “*Low in PSP and/or PA*” set was further supported by proteins with no phosphorylation evidence being enriched for similar functional groups (Figure 5). In fact, we observed a clear decrease in the proportion of proteins enriched for phosphorylation-associated functional groups (where a set was enriched for at least 10 functional groups) going across our established sets suggesting higher phosphosite FDR in lower confidence sets (Figure S5).

**Figure 6:**
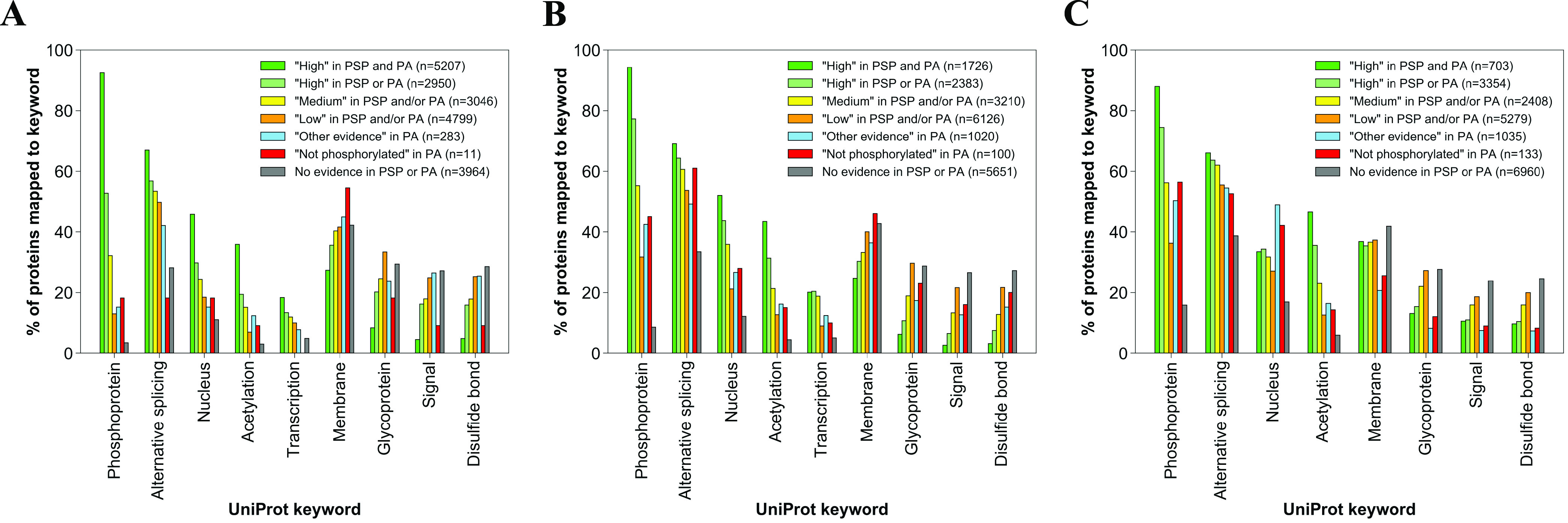
Percentage of proteins within sets containing (A) Ser, (B) Thr, and (C) Tyr sites of various phosphorylation likelihood as their highest-ranked site, annotated with specific UniProt keywords. The number of proteins in each set is presented by *n*.

Our investigation of UniProt terms linked to protein sets revealed that the enrichment for the term “*Phosphoprotein*” and other terms likely to be associated with phosphorylation (“*Alternative splicing*”, “*Nucleus*”, “*Acetylation*”, “*Transcription*”) generally decreased across the sets of reduced confidence, which suggested higher FDR in sets with less phosphorylation evidence (Figure 6). For example, only 13.0, 31.7, and 36.3% of all proteins, which had Ser, Thr, and Tyr sites, respectively, from “*Low in PSP and/or PA*” phosphorylation likelihood set as their most confident site, were marked as phosphoproteins in UniProt (Figure 6 and Table S17), suggesting that most proteins in those sets were not phosphoproteins and further highlighting that the associated sites with only a single piece of evidence are likely false-positive identifications.

### Secondary Structure Analysis

**Figure 7:**
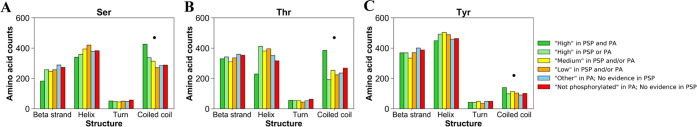
Normalized counts of (A) Ser, (B) Thr, and (C) Tyr amino acids of various phosphorylation likelihood based on evidence in PSP and PA, which are found within protein structures (β strand, α helix, turn, coiled coil). Significant (Fisher’s test *p* value <0.05) enrichment of amino acids from “*High in PSP and PA*” set within protein structures is highlighted by the dot symbol (•) compared with the “*Not phosphorylated*” set.

We also investigated whether Ser, Thr, and Tyr sites with strong phosphorylation evidence were located more frequently within specific protein secondary structures, compared to sites with less evidence. For example, previous analysis of thousands of phosphosites from multiple species identified hotspots within domain families of proteins, particularly near domain interfaces and adjacent to catalytic residues, where they presumably regulate enzymatic output.^65,66^ We found that significantly more (Fisher’s test *p* value <0.05) Ser, Thr, and Tyr sites with the strongest phosphorylation evidence (“*High in PSP and PA*” set) were localized within coiled coils compared to sites in the “*Not phosphorylated*” set (Figure 7). This might readily be explained by coiled coils being frequently found in transcription factors, the activity or subcellular location of which is often dependent on phosphorylation.^67−69^ Therefore, the results in Figure 7 further indicated that there were more potential true Ser, Thr, and Tyr phosphosites in “*High in PSP and PA*” set than in other sets. In terms of other analyzed protein structures (β strand, turn, α helix), there was no significant enrichment of sites from the highest phosphorylation confidence set within those structures compared to the “*Not phosphorylated*” set (Figure 7). In fact, our current reading of the literature suggests that it is still unclear whether phosphorylation sites are found on average to be localized more or less frequently within β strands, turns, or α helices, though clear evidence for localization of PTMs at functionally important loci in proteins has been previously presented.^70^

## Conclusions

In our analysis, we ranked all potential Ser, Thr, and Tyr phosphosites in UniProt reference human proteome according to how much quantitative and qualitative phosphorylation evidence they were assigned in PSP and PA databases. Having analyzed the sites and the proteins that contain them in terms of conservation, proximal site patterns, functional enrichment, and structural properties, we established that Ser, Thr, and Tyr sites with weak phosphosite identification evidence, particularly sites with a single piece of supporting evidence, were likely to be false-positive identifications. This finding was further confirmed by FDR estimations across the established phosphorylation likelihood sets which revealed phosphosite FDR of 84, 98, and 82% in sets of Ser, Thr, and Tyr sites, respectively, where only one piece of identification evidence was present. Since there is a considerable presence of such sites in PSP and PA datasets, our results implied high FDR in both those datasets, although PSP was predicted to have a generally higher proportion of false-positive phosphosites compared to PA. This is potentially a cause for concern since many potential false positives are presented to scientists as true phosphosites, without a clear explanation of the likelihood of such claims. Nevertheless, using our FDR estimates we predicted that there are around 62 000 Ser, 8000 Thr, and 12 000 Tyr true-positive phosphosites in the human proteome that are supported by evidence in PSP and/or PA. These estimated counts are lower than other published estimates^29,30,71,72^ particularly for Ser/Thr sites, presumably due to the previous inclusion of false positives and subsequent overestimation of the number of true phosphosites. We conclude that researchers must be aware of the potential for false-positive sites in both public and self-generated databases and should always evaluate the evidence behind the phosphosites used in their research. As a general rule, phosphorylation sites with <5 independent observations should be treated with caution, and those with only one observation in a database are likely to be false positives. In a recent phosphoproteomics study from our group, we demonstrated the utility of the classification presented here, by matching the sites identified by LC-MS/MS to their evidence categories from PSP and PA.^73^ For new phosphoproteomics studies, it will be common for some ambiguity to remain regarding phosphosite localization, and many sites will be observed that would not pass a 1 or 5% false localization rate cutoff from a single dataset, but for which there may be some supporting evidence. By evaluating new datasets in combination with all of the evidence collated from a large number of previous studies, greater confidence can be assigned to “borderline” significant phosphosites that may indeed be correct, or conversely, sites with weak evidence that have never been reported can be rejected.

Here, we have provided a methodological framework for estimating global FDR in large-scale phosphorylation datasets, which does not rely on native scores from search engines or site localization software. Methods for estimating global FDR in meta-analyses of phosphosites are not yet robust, and thus we would recommend that other groups profile orthogonal properties of ranked sets, as we have done here, to estimate the real distribution of true and false phosphosites in their data.

## Supporting Information Available

Linear regression analysis of conservation within found orthologues between phosphosites and nonphosphosites per protein (Figure S1); proximal site and FDR analysis performed separately for PhosphoSitePlus and PeptideAtlas sets of STY sites (Figure S2); proximal site and FDR analysis of STY sites with phosphorylation evidence in UniProt (Figure S3); count of significant functional groups identified in DAVID for protein sets containing different highest-ranked STY sites (Figure S4); top 10 functional categories for which protein sets containing different highest-ranked STY sites were enriched in DAVID (Figure S5); and proteomes of eukaryotic species used in conservation analysis (Table S3) (PDF)
Filtered PeptideAtlas build with human STY sites that have at least 1 associated PSM (Table S1) (ZIP)
Filtered PhosphoSitePlus build with human STY sites from canonical protein sequences (Table S2) (ZIP)
Proteins in the human proteome that were not analyzed and the reasons for their exclusion (Table S4) (XLSX)
Summary of all STY sites in our analysis, their conservation data, proximal sites, phosphorylation likelihood, and structural data (Table S5) (ZIP)
FASTA sequences of analyzed proteins (Table S6) (ZIP)
Positions of secondary structures within analyzed target proteins in the human proteome (Table S7) (XLSX)
Counts of STY sites in phosphorylation likelihood sets based on evidence in PeptideAtlas before considering the evidence in PhosphoSitePlus (Table S8) (XLSX)
Cross-referencing sets of sites between PhosphoSitePlus and PeptideAtlas (Table S9) (XLSX)
STY sites in human proteome with plenty of phosphorylation evidence in both PhosphoSitePlus and PeptideAtlas (Table S10) (XLSX)
STY conservation scores within proteins that had at least three phosphosites and three nonphosphosites (Table S11) (XLSX)
Conservation of STY sites in each phosphorylation likelihood set (Table S12) (XLSX)
Counts of amino acids adjacent to target STY sites at −1 and +1 positions within phosphorylation likelihood sets (Table S13) (XLSX)
Calculating phosphosite FDR within sets of STY sites ranked according to combined PhosphoSitePlus and PeptideAtlas evidence (Table S14) (XLSX)
Calculating phosphosite FDR within separate PhosphoSitePlus and PeptideAtlas sets of STY sites (Table S15) (XLSX)
Highest-ranked STY site within each analyzed target protein in the human proteome (Table S16) (XLSX)
Percentage of proteins within each ranked set linked to a certain UniProt term (Table S17) (XLSX)

## References

[ref1] Cohen P. The role of protein phosphorylation in human health and disease. The Sir Hans Krebs Medal Lecture. Eur. J. Biochem. 2001, 268, 5001–5010. 10.1046/j.0014-2956.2001.02473.x. 11589691

[ref2] Cohen P. The origins of protein phosphorylation. Nat. Cell Biol. 2002, 4, E127–E130. 10.1038/ncb0502-e127. 11988757

[ref3] Goedert M.; Spillantini M.; Cairns N.; et al. Tau proteins of alzheimer paired helical filaments: Abnormal phosphorylation of all six brain isoforms. Neuron 1992, 8, 159–168. 10.1016/0896-6273(92)90117-V. 1530909

[ref4] Amanchy R.; Kalume D. E.; Iwahori A.; et al. Phosphoproteome Analysis of HeLa Cells Using Stable Isotope Labeling with Amino Acids in Cell Culture (SILAC). J. Proteome Res. 2005, 4, 1661–1671. 10.1021/pr050134h. 16212419

[ref5] Nousiainen M.; Silljé H. H. W.; Sauer G.; et al. Phosphoproteome analysis of the human mitotic spindle. Proc. Natl. Acad. Sci. U.S.A. 2006, 103, 5391 10.1073/pnas.0507066103. 16565220 PMC1459365

[ref6] Olsen J. V.; Blagoev B.; Gnad F.; et al. Global, In Vivo, and Site-Specific Phosphorylation Dynamics in Signaling Networks. Cell 2006, 127, 635–648. 10.1016/j.cell.2006.09.026. 17081983

[ref7] Olsen J. V.; Vermeulen M.; Santamaria A.; et al. Quantitative Phosphoproteomics Reveals Widespread Full Phosphorylation Site Occupancy During Mitosis. Sci. Signaling 2010, 3, ra3 10.1126/scisignal.2000475. 20068231

[ref8] Sharma K.; D’Souza R.; Tyanova S.; et al. Ultradeep human phosphoproteome reveals a distinct regulatory nature of Tyr and Ser/Thr-based signaling. Cell Rep. 2014, 8, 1583–1594. 10.1016/j.celrep.2014.07.036. 25159151

[ref9] Hardman G.; Perkins S.; Brownridge P. J.; et al. Strong anion exchange-mediated phosphoproteomics reveals extensive human non-canonical phosphorylation. EMBO J. 2019, 38, e100847 10.15252/embj.2018100847. 31433507 PMC6826212

[ref10] Alessi D. R.; Andjelkovic M.; Caudwell B.; et al. Mechanism of activation of protein kinase B by insulin and IGF-1. EMBO J. 1996, 15, 6541–6551. 10.1002/j.1460-2075.1996.tb01045.x. 8978681 PMC452479

[ref11] Wettenhall R. E.; Aebersold R. H.; Hood L. E. Solid-phase sequencing of 32P-labeled phosphopeptides at picomole and subpicomole levels. Methods Enzymol. 1991, 201, 186–199. 10.1016/0076-6879(91)01017-v. 1943764

[ref12] Wettenhall R. E.; Erikson E.; Maller J. L. Ordered multisite phosphorylation of Xenopus ribosomal protein S6 by S6 kinase II. J. Biol. Chem. 1992, 267, 9021–9027. 10.1016/S0021-9258(19)50382-9. 1577739

[ref13] Elias J. E.; Gygi S. P. Target-decoy search strategy for increased confidence in large-scale protein identifications by mass spectrometry. Nat. Methods 2007, 4, 207–214. 10.1038/nmeth1019. 17327847

[ref14] Käll L.; Storey J. D.; Noble W. S. QVALITY: non-parametric estimation of q-values and posterior error probabilities. Bioinformatics 2009, 25, 964–966. 10.1093/bioinformatics/btp021. 19193729 PMC2660870

[ref15] Söderholm S.; Hintsanen P.; Öhman T.; et al. PhosFox: a bioinformatics tool for peptide-level processing of LC-MS/MS-based phosphoproteomic data. Proteome Sci. 2014, 12, 36 10.1186/1477-5956-12-36. 25028575 PMC4098950

[ref16] Lee D. C. H.; Jones A. R.; Hubbard S. J. Computational phosphoproteomics: from identification to localization. Proteomics 2015, 15, 950–963. 10.1002/pmic.201400372. 25475148 PMC4384807

[ref17] Wang Y.; Tian Y.; Liu X.; et al. A New Workflow for the Analysis of Phosphosite Occupancy in Paired Samples by Integration of Proteomics and Phosphoproteomics Data Sets. J. Proteome Res. 2020, 19, 3807–3816. 10.1021/acs.jproteome.0c00345. 32786891

[ref18] Taus T.; Köcher T.; Pichler P.; et al. Universal and confident phosphorylation site localization using phosphoRS. J. Proteome Res. 2011, 10, 5354–5362. 10.1021/pr200611n. 22073976

[ref19] Beausoleil S. A.; Villén J.; Gerber S. A.; et al. A probability-based approach for high-throughput protein phosphorylation analysis and site localization. Nat. Biotechnol. 2006, 24, 1285–1292. 10.1038/nbt1240. 16964243

[ref20] Cox J.; Neuhauser N.; Michalski A.; et al. Andromeda: a peptide search engine integrated into the MaxQuant environment. J. Proteome Res. 2011, 10, 1794–1805. 10.1021/pr101065j. 21254760

[ref21] Shteynberg D. D.; Deutsch E. W.; Campbell D. S.; et al. PTMProphet: Fast and Accurate Mass Modification Localization for the Trans-Proteomic Pipeline. J. Proteome Res. 2019, 18, 4262–4272. 10.1021/acs.jproteome.9b00205. 31290668 PMC6898736

[ref22] Ferries S.; Perkins S.; Brownridge P. J.; et al. Evaluation of Parameters for Confident Phosphorylation Site Localization Using an Orbitrap Fusion Tribrid Mass Spectrometer. J. Proteome Res. 2017, 16, 3448–3459. 10.1021/acs.jproteome.7b00337. 28741359

[ref23] Dephoure N.; Gould K. L.; Gygi S. P.; et al. Mapping and analysis of phosphorylation sites: a quick guide for cell biologists. Mol. Biol. Cell 2013, 24, 535–542. 10.1091/mbc.e12-09-0677. 23447708 PMC3583658

[ref24] Chalkley R. J.; Clauser K. R. Modification site localization scoring: strategies and performance. Mol. Cell. Proteomics 2012, 11, 3–14. 10.1074/mcp.R111.015305. 22328712 PMC3418845

[ref25] Hoopmann M. R.; Kusebauch U.; Palmblad M.; et al. Insights from the First Phosphopeptide Challenge of the MS Resource Pillar of the HUPO Human Proteome Project. J. Proteome Res. 2020, 19, 4754–4765. 10.1021/acs.jproteome.0c00648. 33166149 PMC8204901

[ref26] Desiere F.; et al. The PeptideAtlas project. Nucleic Acids Res. 2006, 34, D655–D658. 10.1093/nar/gkj040. 16381952 PMC1347403

[ref27] Dinkel H.; Chica C.; Via A.; et al. Phospho.ELM: a database of phosphorylation sites--update 2011. Nucleic Acids Res. 2011, 39, D261–D267. 10.1093/nar/gkq1104. 21062810 PMC3013696

[ref28] Gnad F.; Gunawardena J.; Mann M. PHOSIDA 2011: the posttranslational modification database. Nucleic Acids Res. 2011, 39, D253–D260. 10.1093/nar/gkq1159. 21081558 PMC3013726

[ref29] Hornbeck P. V.; Zhang B.; Murray B.; et al. PhosphoSitePlus, 2014: mutations, PTMs and recalibrations. Nucleic Acids Res. 2015, 43, D512–D520. 10.1093/nar/gku1267. 25514926 PMC4383998

[ref30] Ochoa D.; Jarnuczak A. F.; Viéitez C.; et al. The functional landscape of the human phosphoproteome. Nat. Biotechnol. 2020, 38, 365–373. 10.1038/s41587-019-0344-3. 31819260 PMC7100915

[ref31] UniProt: a worldwide hub of protein knowledge. Nucleic Acids Res. 2019, 47, D506–D515. 10.1093/nar/gky1049. 30395287 PMC6323992

[ref32] Deutsch E. W.; Mendoza L.; Shteynberg D.; et al. A guided tour of the Trans-Proteomic Pipeline. Proteomics 2010, 10, 1150–1159. 10.1002/pmic.200900375. 20101611 PMC3017125

[ref33] Huang K.-Y.; Lee T. Y.; Kao H. J.; et al. dbPTM in 2019: exploring disease association and cross-talk of post-translational modifications. Nucleic Acids Res. 2019, 47, D298–D308. 10.1093/nar/gky1074. 30418626 PMC6323979

[ref34] Giansanti P.; Aye T.; van den Toorn H.; et al. An Augmented Multiple-Protease-Based Human Phosphopeptide Atlas. Cell Rep. 2015, 11, 1834–1843. 10.1016/j.celrep.2015.05.029. 26074081

[ref35] Altschul S. F.; Gish W.; Miller W.; et al. Basic local alignment search tool. J. Mol. Biol. 1990, 215, 403–410. 10.1016/S0022-2836(05)80360-2. 2231712

[ref36] Edgar R. C. MUSCLE: multiple sequence alignment with high accuracy and high throughput. Nucleic Acids Res. 2004, 32, 1792–1797. 10.1093/nar/gkh340. 15034147 PMC390337

[ref37] Fisher R. A. Statistical Methods for Research Workers. In Breakthroughs in Statistics: Methodology and Distribution, Kotz S.; Johnson N. L., Eds.; Springer: New York, 1992; pp 66–70.

[ref38] Virtanen P.; Gommers R.; Oliphant T. E.; et al. SciPy 1.0: fundamental algorithms for scientific computing in Python. Nat. Methods 2020, 17, 261–272. 10.1038/s41592-019-0686-2. 32015543 PMC7056644

[ref39] DennisG.; ShermanB. T.; HosackD. A.; et al. DAVID: Database for Annotation, Visualization, and Integrated Discovery. Genome Biol. 2003, 4, P310.1186/gb-2003-4-5-p3.12734009

[ref40] Rikova K.; Guo A.; Zeng Q.; et al. Global survey of phosphotyrosine signaling identifies oncogenic kinases in lung cancer. Cell 2007, 131, 1190–1203. 10.1016/j.cell.2007.11.025. 18083107

[ref41] Rush J.; Moritz A.; Lee K. A.; et al. Immunoaffinity profiling of tyrosine phosphorylation in cancer cells. Nat. Biotechnol. 2005, 23, 94–101. 10.1038/nbt1046. 15592455

[ref42] Boekhorst J.; van Breukelen B.; Heck A. J.; et al. Comparative phosphoproteomics reveals evolutionary and functional conservation of phosphorylation across eukaryotes. Genome Biol. 2008, 9, R144 10.1186/gb-2008-9-10-r144. 18828897 PMC2760871

[ref43] Malik R.; Nigg E. A.; Körner R. Comparative conservation analysis of the human mitotic phosphoproteome. Bioinformatics 2008, 24, 1426–1432. 10.1093/bioinformatics/btn197. 18426804

[ref44] Studer R. A.; Rodriguez-Mias R. A.; Haas K. M.; et al. Evolution of protein phosphorylation across 18 fungal species. Science 2016, 354, 229–232. 10.1126/science.aaf2144. 27738172

[ref45] Chen S. C.-C.; Chen F.-C.; Li W.-H. Phosphorylated and nonphosphorylated serine and threonine residues evolve at different rates in mammals. Mol. Biol. Evol. 2010, 27, 2548–2554. 10.1093/molbev/msq142. 20534707 PMC2955733

[ref46] Landry C. R.; Levy E. D.; Michnick S. W. Weak functional constraints on phosphoproteomes. Trends Genet. 2009, 25, 193–197. 10.1016/j.tig.2009.03.003. 19349092

[ref47] Lienhard G. E. Non-functional phosphorylations?. Trends Biochem. Sci. 2008, 33, 351–352. 10.1016/j.tibs.2008.05.004. 18603430

[ref48] Byrne D. P.; Clarke C. J.; Brownridge P. J.; et al. Use of the Polo-like kinase 4 (PLK4) inhibitor centrinone to investigate intracellular signalling networks using SILAC-based phosphoproteomics. Biochem. J. 2020, 477, 2451–2475. 10.1042/BCJ20200309. 32501498 PMC7338032

[ref49] Hutti J. E.; Jarrell E. T.; Chang J. D.; et al. A rapid method for determining protein kinase phosphorylation specificity. Nat. Methods 2004, 1, 27–29. 10.1038/nmeth708. 15782149

[ref50] Kettenbach A. N.; Wang T.; Faherty B.; et al. Rapid determination of multiple linear kinase substrate motifs by mass spectrometry. Chem. Biol. 2012, 19, 608–618. 10.1016/j.chembiol.2012.04.011. 22633412 PMC3366114

[ref51] Hall F. L.; Vulliet P. R. Proline-directed protein phosphorylation and cell cycle regulation. Curr. Opin. Cell Biol. 1991, 3, 176–184. 10.1016/0955-0674(91)90136-M. 1831990

[ref52] Johnson L. N.; Lowe E.; Noble M.; et al. The Eleventh Datta Lecture. The structural basis for substrate recognition and control by protein kinases. FEBS Lett. 1998, 430, 1–11. 10.1016/S0014-5793(98)00606-1. 9678585

[ref53] Keshwani M. M.; et al. Conserved proline-directed phosphorylation regulates SR protein conformation and splicing function. Biochem. J. 2015, 466, 311–322. 10.1042/BJ20141373. 25529026 PMC5053020

[ref54] Lu K. P.; Liou Y.-C.; Zhou X. Z. Pinning down proline-directed phosphorylation signaling. Trends Cell Biol. 2002, 12, 164–172. 10.1016/S0962-8924(02)02253-5. 11978535

[ref55] Pietrangelo A.; Ridgway N. D. Phosphorylation of a serine/proline-rich motif in oxysterol binding protein-related protein 4L (ORP4L) regulates cholesterol and vimentin binding. PLoS One 2019, 14, e0214768 10.1371/journal.pone.0214768. 30925160 PMC6440634

[ref56] Songyang Z.; Blechner S.; Hoagland N.; et al. Use of an oriented peptide library to determine the optimal substrates of protein kinases. Curr. Biol. 1994, 4, 973–982. 10.1016/S0960-9822(00)00221-9. 7874496

[ref57] Amanchy R.; Periaswamy B.; Mathivanan S.; et al. A curated compendium of phosphorylation motifs. Nat. Biotechnol. 2007, 25, 285–286. 10.1038/nbt0307-285. 17344875

[ref58] Sugiyama N.; Imamura H.; Ishihama Y. Large-scale Discovery of Substrates of the Human Kinome. Sci. Rep. 2019, 9, 10503 10.1038/s41598-019-46385-4. 31324866 PMC6642169

[ref59] Songyang Z.; Lu K. P.; Kwon Y. T.; et al. A structural basis for substrate specificities of protein Ser/Thr kinases: primary sequence preference of casein kinases I and II, NIMA, phosphorylase kinase, calmodulin-dependent kinase II, CDK5, and Erk1. Mol. Cell. Biol. 1996, 16, 6486–6493. 10.1128/MCB.16.11.6486. 8887677 PMC231650

[ref60] Wälchli S.; Espanel X.; Harrenga A.; et al. Probing protein-tyrosine phosphatase substrate specificity using a phosphotyrosine-containing phage library. J. Biol. Chem. 2004, 279, 311–318. 10.1074/jbc.M307617200. 14578355

[ref61] Espinos E.; Thaï A. L. V.; Pomiès C.; et al. Cooperation between phosphorylation and acetylation processes in transcriptional control. Mol. Cell. Biol. 1999, 19, 3474–3484. 10.1128/MCB.19.5.3474. 10207071 PMC84140

[ref62] Habibian J.; Ferguson B. S. The Crosstalk between Acetylation and Phosphorylation: Emerging New Roles for HDAC Inhibitors in the Heart. Int. J. Mol. Sci. 2019, 20, 102 10.3390/ijms20010102. PMC6337125 30597863

[ref63] Leney A. C.; El Atmioui D.; Wu W.; et al. Elucidating crosstalk mechanisms between phosphorylation and O-GlcNAcylation. Proc. Natl. Acad. Sci. U.S.A. 2017, 114, E7255–E7261. 10.1073/pnas.1620529114. 28808029 PMC5584407

[ref64] Naro C.; Sette C. Phosphorylation-mediated regulation of alternative splicing in cancer. Int. J. Cell Biol. 2013, 2013, 151839 10.1155/2013/151839. 24069033 PMC3771450

[ref65] Beltrao P.; Albanèse V.; Kenner L.; et al. Systematic functional prioritization of protein posttranslational modifications. Cell 2012, 150, 413–425. 10.1016/j.cell.2012.05.036. 22817900 PMC3404735

[ref66] Strumillo M. J.; Oplová M.; Viéitez C.; et al. Conserved phosphorylation hotspots in eukaryotic protein domain families. Nat. Commun. 2019, 10, 1977 10.1038/s41467-019-09952-x. 31036831 PMC6488607

[ref67] Barbara K. E.; Willis K. A.; Haley T. M.; et al. Coiled coil structures and transcription: an analysis of the S. cerevisiae coilome. Mol. Genet. Genomics 2007, 278, 135–147. 10.1007/s00438-007-0237-x. 17476531

[ref68] Baxevanis A. D.; Vinson C. R. Interactions of coiled coils in transcription factors: where is the specificity?. Curr. Opin. Genet. Dev. 1993, 3, 278–285. 10.1016/0959-437X(93)90035-N. 8504253

[ref69] Pogenberg V.; Ballesteros-Álvarez J.; Schober R.; et al. Mechanism of conditional partner selectivity in MITF/TFE family transcription factors with a conserved coiled coil stammer motif. Nucleic Acids Res. 2020, 48, 934–948. 10.1093/nar/gkz1104. 31777941 PMC6954422

[ref70] Gu G. M.; Wang J. K. [DNA-binding profiles of mammalian transcription factors]. Yi Chuan 2012, 34, 950–968. 10.3724/SP.J.1005.2012.00950. 22917900

[ref71] Keegan S.; Cortens J. P.; Beavis R. C.; et al. g2pDB: A Database Mapping Protein Post-Translational Modifications to Genomic Coordinates. J. Proteome Res. 2016, 15, 983–990. 10.1021/acs.jproteome.5b01018. 26842767

[ref72] Safaei J.; Maňuch J.; Gupta A.; et al. Prediction of 492 human protein kinase substrate specificities. Proteome Sci. 2011, 9, S6 10.1186/1477-5956-9-S1-S6. 22165948 PMC3379035

[ref73] Campbell A. E.; Ferraz Franco C.; Su L. I.; et al. Temporal modulation of the NF-κB RelA network in response to different types of DNA damage. Biochem. J. 2021, 478, 533–551. 10.1042/BCJ20200627. 33438746 PMC7886319

