# Supplementary material for: Profiling the Human Phosphoproteome to Estimate the True Extent of Protein Phosphorylation
Source: J Proteome Res. 2022 May 9;21(6):1510–24. doi: 10.1021/acs.jproteome.2c00131 (PMC9171898; doi:10.1021/acs.jproteome.2c00131)
Supplement: Supplementary file 1 — pr2c00131_si_001.pdf [file pr2c00131_si_001.pdf]

Profiling the Human Phosphoproteome to Estimate the True Extent of Protein Phosphorylation

Anton Kalyuzhnyy[1,2], Patrick A. Eyers[1], Claire E. Eyers[1,3], Emily Bowler-Barnett[4], Maria J. Martin[4], Zhi Sun[5], Eric W. Deutsch[5] and Andrew R. Jones*[1,2] (*andrew.jones@liverpool.ac.uk*)

[1]Department of Biochemistry and Systems Biology, Institute of Systems, Molecular and Integrative Biology, University of Liverpool, Liverpool, UK

[2]Computational Biology Facility, Department of Biochemistry and Systems Biology, Institute of Systems, Molecular and Integrative Biology, University of Liverpool, Liverpool, UK

[3]Centre for Proteome Research, Department of Biochemistry and Systems Biology, Institute of Systems, Molecular and Integrative Biology, University of Liverpool, Liverpool, UK

[4]European Molecular Biology Laboratory, European Bioinformatics Institute (EMBL-EBI), Cambridge, United Kingdom

[5]Institute for Systems Biology, Seattle, Washington 98109, United States

Supplementary information

**Figure S1.** Linear regression analysis of conservation within found orthologues between phosphosites and non-phosphosites per protein.	Page S-1
**Figure S2.** Proximal site and FDR analysis performed separately for PhosphoSitePlus and PeptideAtlas sets of STY sites.	Page S-2
**Figure S3.** Proximal site and FDR analysis of STY sites with phosphorylation evidence in UniProt.	Pages S-3, S4
**Figure S4.** Count of significant functional groups identified in DAVID for protein sets containing different highest ranked STY sites.	Page S-5
**Figure S5.** Top 10 functional categories for which protein sets containing different highest ranked STY sites were enriched in DAVID.	Page S-6
**Table S1.** Filtered PeptideAtlas build with human STY sites that have at least 1 associated PSM.	Submitted separately as .zip file
**Table S2.** Filtered PhosphoSitePlus build with human STY sites from canonical protein sequences.	Submitted separately as .zip file
**Table S3.** Proteomes of eukaryotic species used in conservation analysis.	Pages S-7, S-8, S-9
**Table S4.** Proteins in the human proteome which were not analysed and the reasons for their exclusion.	Submitted separately as .xlsx file
**Table S5.** Summary of all STY sites in our analysis, their conservation data, proximal sites, phosphorylation likelihood and structural data.	Submitted separately as .zip file
**Table S6.** FASTA sequences of analysed proteins.	Submitted separately as .zip file
**Table S7.** Positions of secondary structures within analysed target proteins in the human proteome.	Submitted separately as .xlsx file
**Table S8.** Counts of STY sites in phosphorylation likelihood sets based on evidence in PeptideAtlas before considering evidence in PhosphoSitePlus.	Submitted separately as .xlsx file
**Table S9.** Cross-referencing sets of sites between PhosphoSitePlus and PeptideAtlas	Submitted separately as .xlsx file
**Table S10.** STY sites in human proteome with plenty of phosphorylation evidence in both PhosphoSitePlus and PeptideAtlas.	Submitted separately as .xlsx file
**Table S11.** STY conservation scores within proteins which had at least 3 phosphosites and 3 non-phosphosites.	Submitted separately as .xlsx file
**Table S12.** Conservation of STY sites in each phosphorylation likelihood set.	Submitted separately as .xlsx file
**Table S13.** Counts of amino acids adjacent to target STY sites at -1 and +1 positions within phosphorylation likelihood sets.	Submitted separately as .xlsx file
**Table S14.** Calculating phosphosite FDR within sets of STY sites ranked according to combined PhosphoSitePlus and PeptideAtlas evidence.	Submitted separately as .xlsx file
**Table S15.** Calculating phosphosite FDR within separate PhosphoSitePlus and PeptideAtlas sets of STY sites.	Submitted separately as .xlsx file
**Table S16.** Highest ranked STY site within each analysed target protein in the human proteome.	Submitted separately as .xlsx file
**Table S17.** Percentage of proteins within each ranked set linked to a certain UniProt term.	Submitted separately as .xlsx file

A
B
C
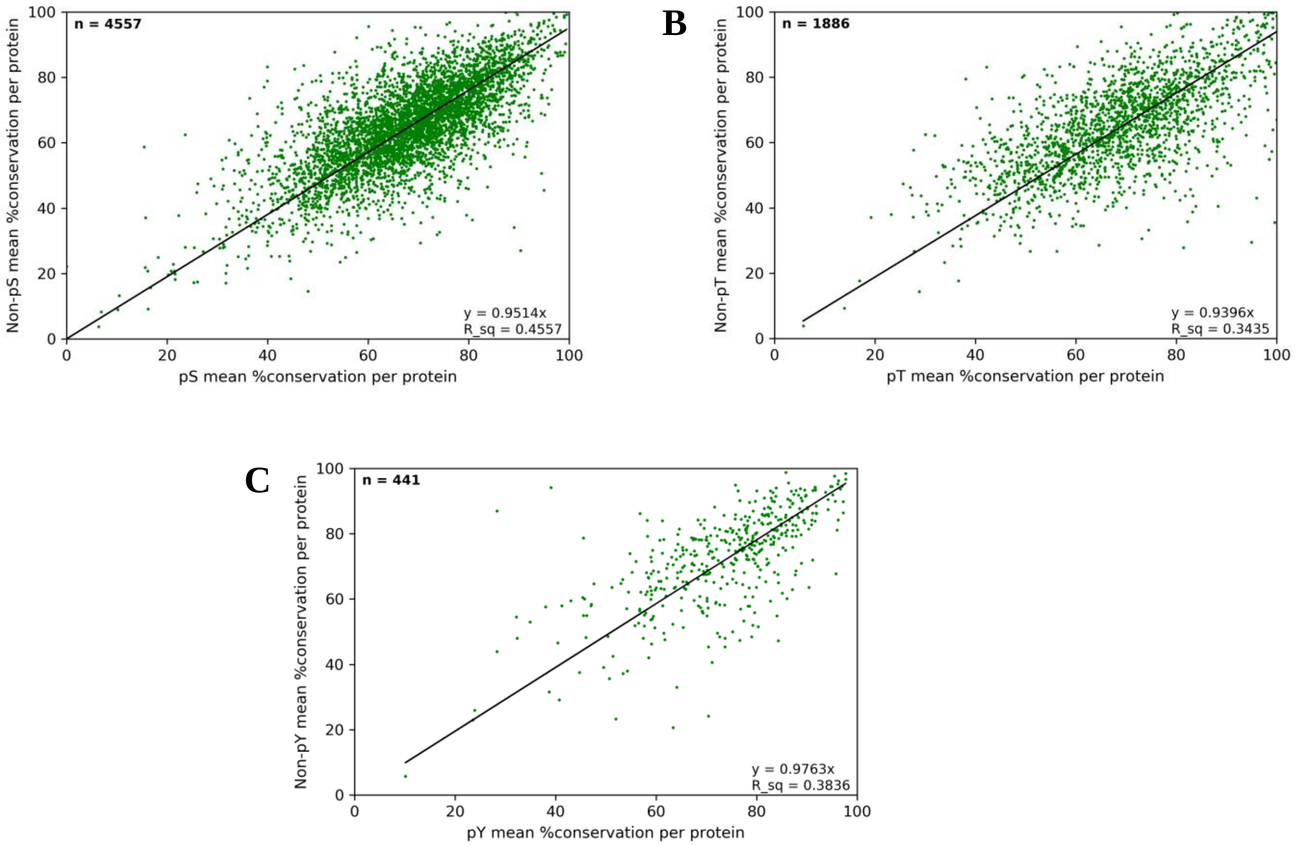

Figure S1. Mean % conservation across found orthologues of likely **(A)** Ser, (**B)** Thr, (**C)** Tyr phosphosites and corresponding likely non-phosphosites per each human protein in the analysed set (n). The R^2 coefficient is given by 'R_sq'.

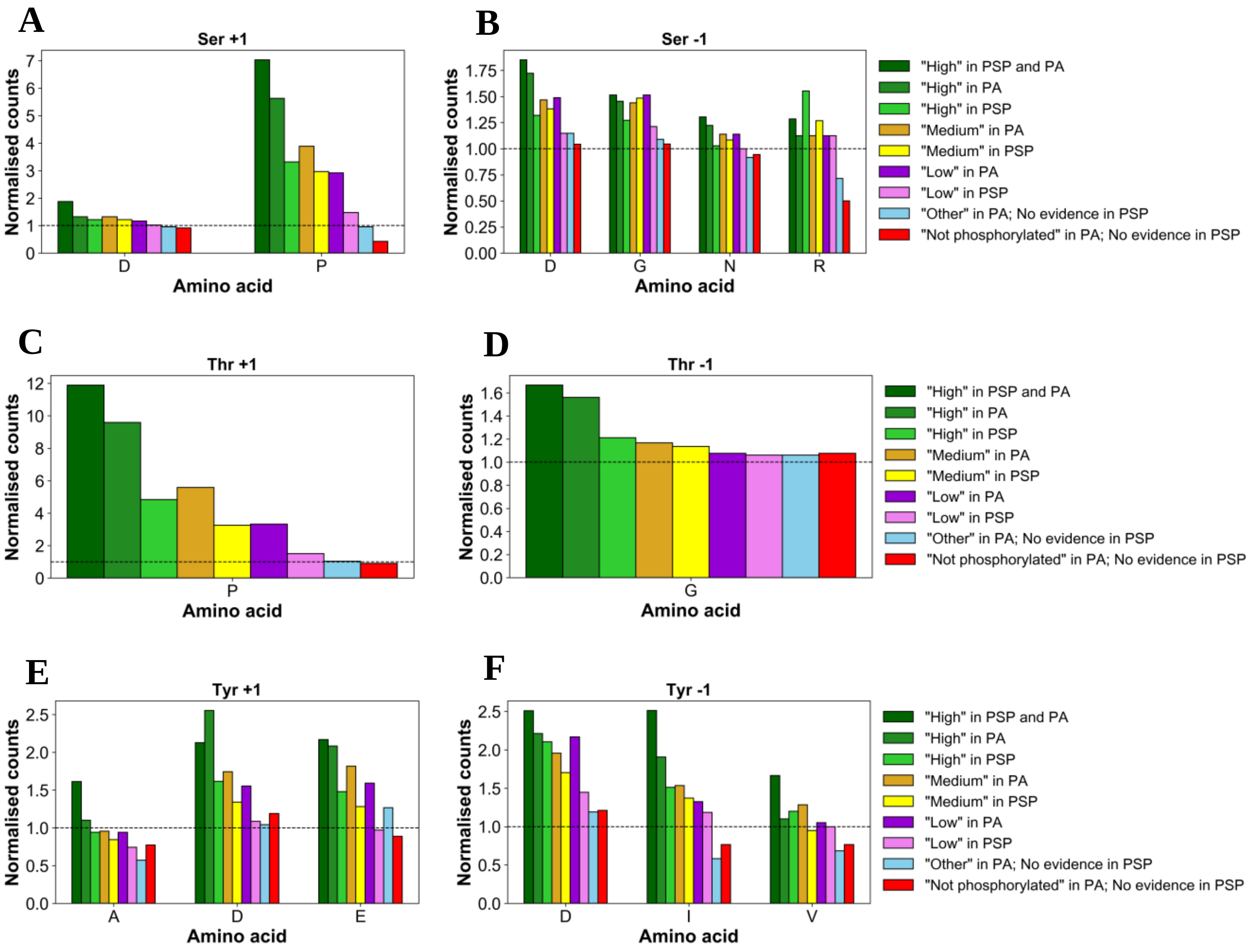

Figure S2. Normalised counts of proximal amino acids positioned at **(A)** +1 around Ser; **(B)** -1 around Ser; **(C)** +1 around Thr; **(D)** -1 around Thr; **(E)** +1 around Tyr; **(F)** -1 around Tyr sites of various phosphorylation likelihood based on evidence in PSP and PA, which are significantly (Bonferroni corrected p value < 0.001) enriched in the "*High" in PSP and PA*" compared to the "*Not phosphorylated*" set and to the expected amino acid distribution in the human proteome (represented by dotted baseline).

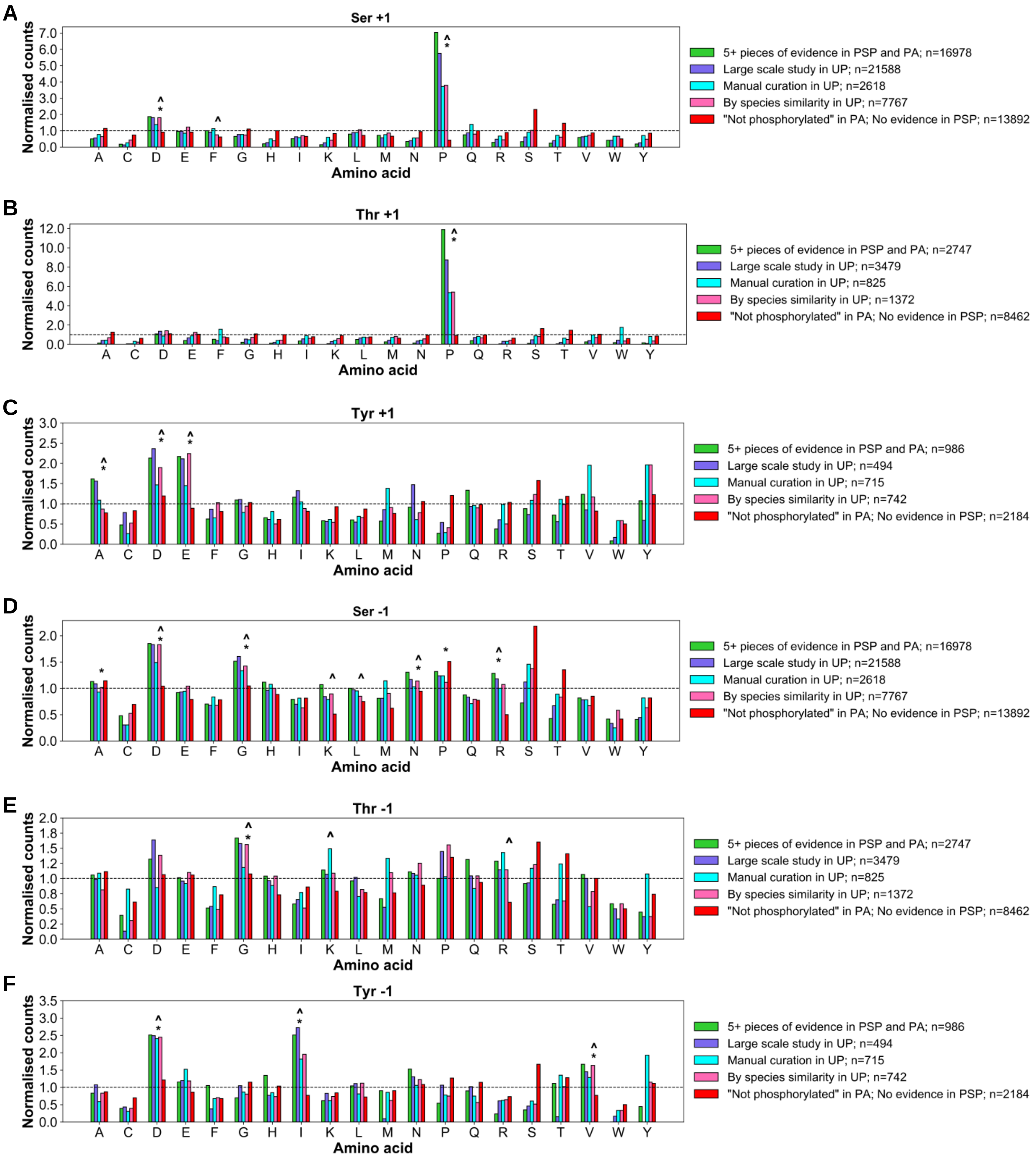

G

Evidence source in UniProt	Ser count	Ser % FDR (95% CI)	Ser TP count	Thr count	Thr % FDR (95% CI)	Thr TP count	Tyr count	Tyr % FDR (95% CI)	Tyr TP count
Large scale study data	21588	7 (± 8)	20077	3479	22 (± 14)	3910	494	6 (± 7)	464

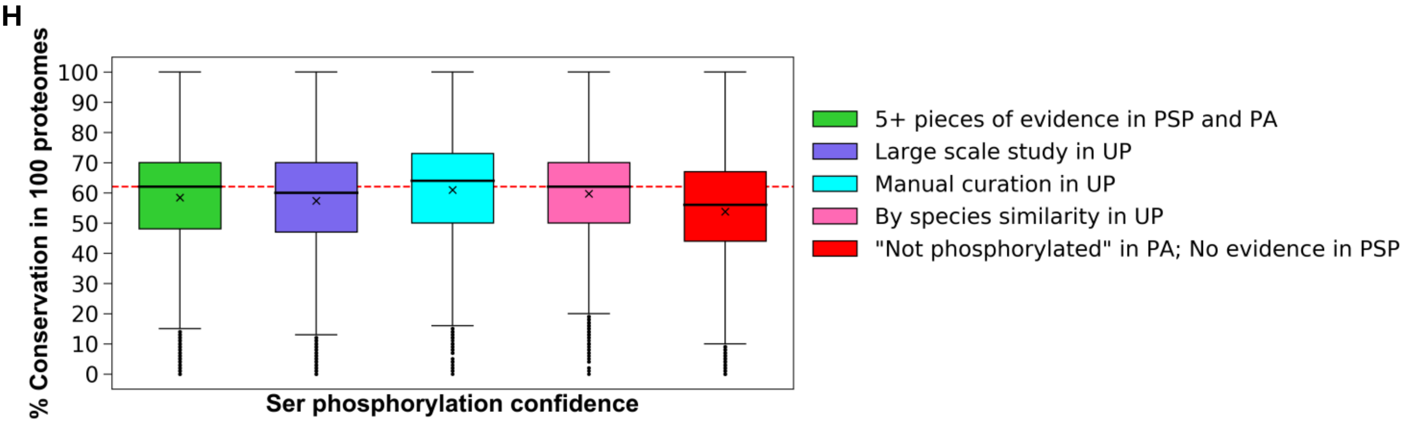

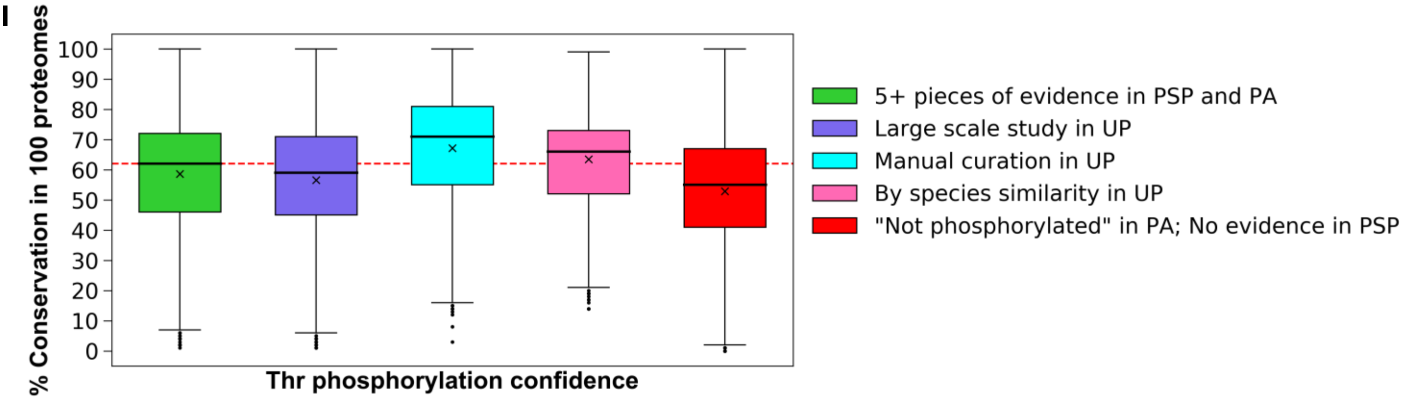

Figure S3. The analysis of UniProt (UP) sites. Counts of proximal amino acids positioned at **(A)** +1 around Ser; **(B)** +1 around Thr; **(C)** +1 around Tyr; **(D)** -1 around Ser; **(E)** -1 around Thr; **(F)** -1 around Tyr sites with different phosphorylation evidence types across PhosphoSitePlus (PSP), PeptideAtlas (PA) and UP, normalised to observed distribution of those amino acids in human proteome (represented by dotted baseline fixed at 1). Significant (Bonferroni corrected p value <0.001) enrichment of proximal amino acids in the "*5+ pieces of evidence in PSP and PA*" set is highlighted by the caret symbol (^) when compared against the "*Not phosphorylated*" set, and an asterisk symbol (*) when compared to the expected amino acid distribution. **(G)** False discovery rate (FDR) and counts of estimated true positive (TP) Ser, Thr and Tyr phosphosites within a set of UniProt sites identified by large scale proteomics studies and which are found in the reference human proteome. Box plots show conservation percentages (%) across 100 eukaryotic species of human **(H)** Ser, **(I)** Thr sites categorised based on evidence type in PSP/PA and UniProt (UP). Within each box, a horizontal line represents median % conservation, an (x) symbol represents mean % conservation per group. Each box extends from the 25th to the 75th percentile of each set's distribution of conservation % values. Vertical lines extending from the boxes correspond to adjacent values. Dots (•) represent outlier values. Red line shows median % conservation in "*5+ pieces of evidence in PSP and PA*" set for visual comparison.

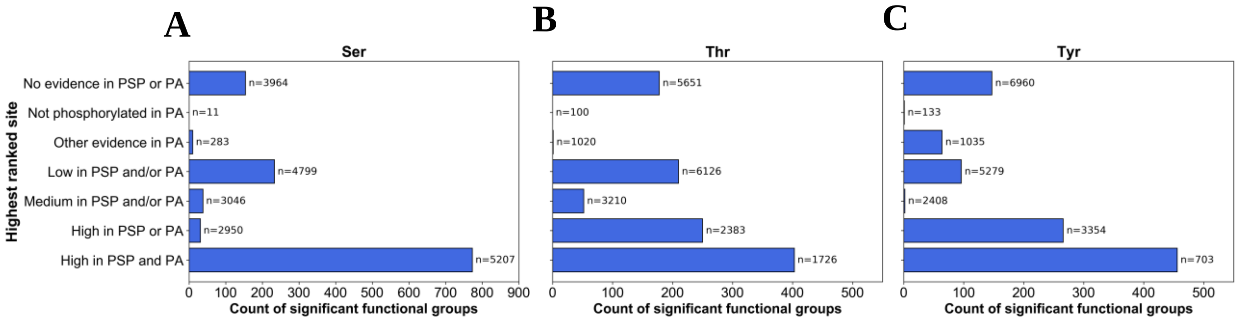

Figure S4. Count of significant (Benjamini–Hochberg adj. p value <0.05) functional groups identified in DAVID for protein sets containing different highest ranked **(A)** Ser, **(B)** Thr, **(C)** Tyr sites based on phosphorylation likelihood sets in PSP and PA. The number of proteins in each set is presented by n.

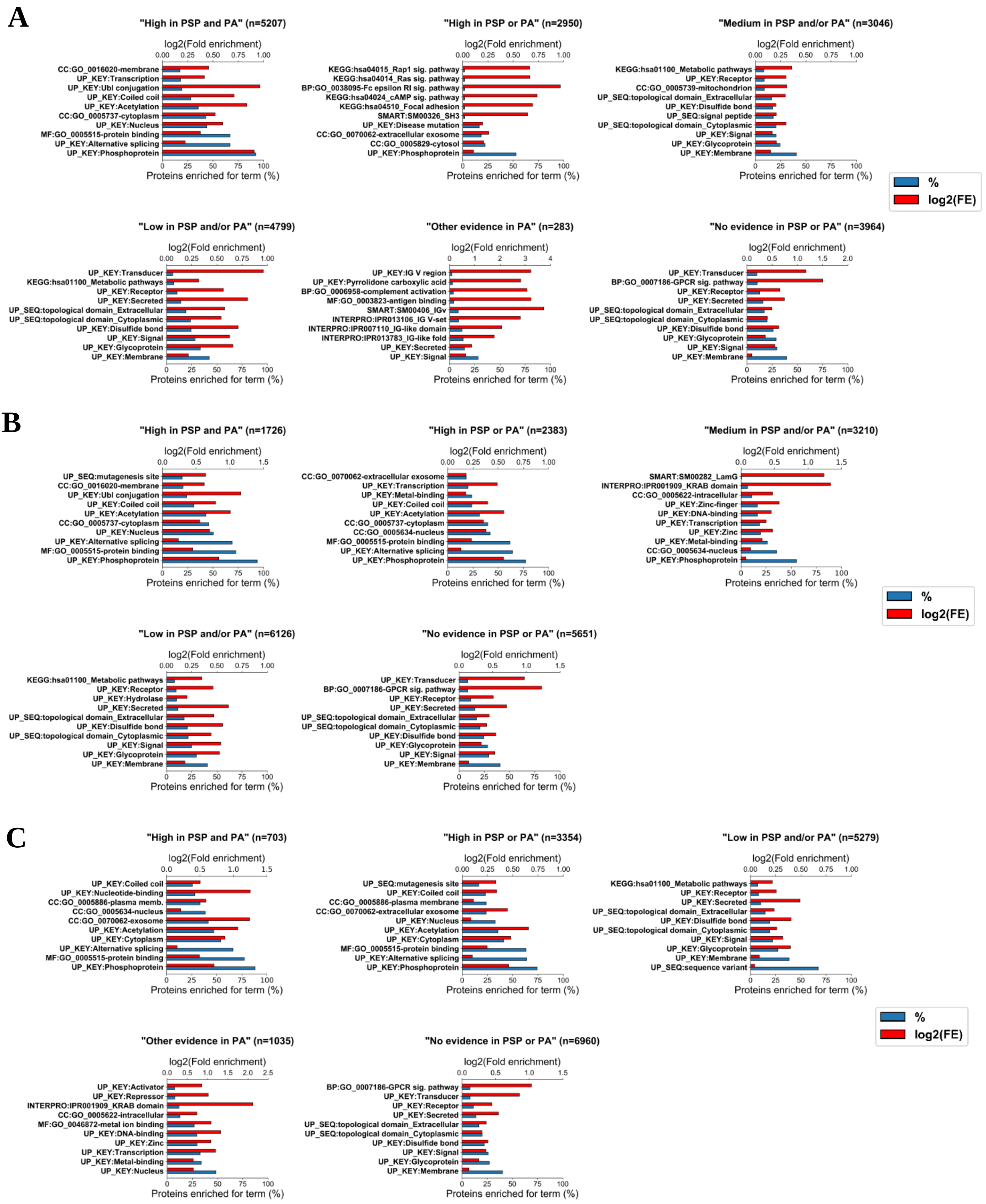

Figure S5. Top 10 functional categories for which protein sets containing various highest ranked **(A)** Ser, **(B)** Thr, **(C)** Tyr sites based on the amount of available phosphorylation evidence were significantly enriched in DAVID (Benjamini–Hochberg corrected p value <0.05). For each protein set, the percentage of proteins (%) enriched for a particular functional category is given as well as the log2(fold enrichment) for that category. The number of proteins in each set is presented by n.

Table S3. Proteomes of eukaryotic species analysed in the conservation analysis.

Organism name from Uniprot	Proteome ID	Organism ID	BUSCO score	Gene count
Pan troglodytes (Chimpanzee)	UP000002277	9598	C:98.3%[S:48.4%,D:49.9%],F:0.5%,M:1.1%,n:6192	23003
Pan paniscus (Pygmy chimpanzee)	UP000240080	9597	C:97.4%[S:54.4%,D:43%],F:1.4%,M:1.1%,n:6192	21211
Gorilla gorilla gorilla (Western lowland gorilla)	UP000001519	9595	C:97.3%[S:52%,D:45.3%],F:1.8%,M:1%,n:6192	21787
Pongo abelii (Sumatran orangutan)	UP000001595	9601	C:94.5%[S:88.5%,D:6%],F:4.2%,M:1.3%,n:6192	21992
Nomascus leucogenys (Northern white-cheeked gibbon)	UP000001073	61853	C:96.3%[S:57.7%,D:38.6%],F:2.6%,M:1.1%,n:6192	20753
Macaca mulatta (Rhesus macaque)	UP000006718	9544	C:89.6%[S:47.8%,D:41.8%],F:1.5%,M:8.9%,n:6192	21868
Cercocebus atys (Sooty mangabey)	UP000233060	9531	C:98%[S:48.3%,D:49.7%],F:1.1%,M:0.9%,n:6192	20874
Rhinopithecus bieti (Black snub-nosed monkey)	UP000233180	61621	C:95.1%[S:49.6%,D:45.5%],F:2.9%,M:2%,n:6192	20845
Chlorocebus sabaeus (Green monkey)	UP000029965	60711	C:95.4%[S:94.4%,D:1%],F:3.4%,M:1.2%,n:6192	19136
Macaca fascicularis (Crab-eating macaque)	UP000233100	9541	C:98.2%[S:48.8%,D:49.3%],F:1.1%,M:0.8%,n:6192	22278
Papio anubis (Olive baboon)	UP000028761	9555	C:98.4%[S:51.7%,D:46.7%],F:0.8%,M:0.8%,n:6192	21559
Mandrillus leucophaeus (Drill)	UP000233140	9568	C:95.5%[S:55.1%,D:40.3%],F:3%,M:1.6%,n:6192	20767
Saimiri boliviensis boliviensis (Bolivian squirrel monkey)	UP000233220	39432	C:96.3%[S:50.1%,D:46.2%],F:2.2%,M:1.5%,n:6192	19356
Callithrix jacchus (White-tufted-ear marmoset)	UP000008225	9483	C:97.9%[S:52.7%,D:45.2%],F:1%,M:1.1%,n:6192	22587
Aotus nancymaae (Ma's night monkey)	UP000233020	37293	C:97.4%[S:50.9%,D:46.5%],F:1.3%,M:1.3%,n:6192	20363
Tarsius syrichta (Philippine tarsier)	UP000189704	1868482	C:75.6%[S:60%,D:15.6%],F:5.7%,M:18.7%,n:6192	19956
Otolemur garnettii (Small-eared galago)	UP000005225	30611	C:96.8%[S:94%,D:2.8%],F:2.1%,M:1.1%,n:6192	19443
Propithecus coquereli (Coquerel's sifaka)	UP000233160	379532	C:92%[S:63.6%,D:28.4%],F:4.1%,M:3.9%,n:6192	17876
Ictidomys tridecemlineatus (Thirteen-lined ground squirrel)	UP000005215	43179	C:94.2%[S:71.1%,D:23%],F:2.6%,M:3.2%,n:6192	18440
Cavia porcellus (Guinea pig)	UP000005447	10141	C:94.2%[S:70.3%,D:23.9%],F:2.9%,M:2.9%,n:6192	18247
Mus musculus (Mouse)	UP000000589	10090	C:99.7%[S:52.1%,D:47.6%],F:0.2%,M:0.1%,n:6192	21982
Oryctolagus cuniculus (Rabbit)	UP000001811	9986	C:91.2%[S:84.3%,D:6.9%],F:4.6%,M:4.1%,n:6192	21178
Cricetulus griseus (Chinese hamster)	UP000001075	10029	C:63.7%[S:63%,D:0.7%],F:19.2%,M:17.1%,n:6192	23874
Fukomys damarensis (Damaraland mole rat)	UP000028990	885580	C:82.4%[S:81.9%,D:0.5%],F:7.7%,M:9.9%,n:6192	20401
Mesocricetus auratus (Golden hamster)	UP000189706	10036	C:77.6%[S:54.7%,D:22.9%],F:1.9%,M:20.5%,n:6192	20418
Dipodomys ordii (Ord's kangaroo rat)	UP000081671	10020	C:93.7%[S:66.1%,D:27.6%],F:5.4%,M:0.9%,n:6192	19730
Heterocephalus glaber (Naked mole rat)	UP000006813	10181	C:87%[S:85.5%,D:1.5%],F:6.7%,M:6.2%,n:6192	21445
Vombatus ursinus (Common wombat)	UP000314987	29139	C:96.6%[S:56.2%,D:40.4%],F:1.8%,M:1.6%,n:4104	19872
Myotis lucifugus (Little brown bat)	UP000001074	59463	C:95.1%[S:90%,D:5.1%],F:3.5%,M:1.4%,n:6253	19655
Canis lupus familiaris (Dog)	UP000002254	9615	C:97%[S:45.5%,D:51.5%],F:1.7%,M:1.3%,n:6253	20624
Capra hircus (Goat)	UP000291000	9925	C:98%[S:57.9%,D:40.1%],F:1.2%,M:0.8%,n:6253	21149
Ovis aries (Sheep)	UP000002356	9940	C:98%[S:86.8%,D:11.2%],F:1.4%,M:0.5%,n:6253	21212
Sus scrofa (Pig)	UP000008227	9823	C:95.3%[S:49%,D:46.3%],F:2.4%,M:2.3%,n:6253	22130
Felis catus (Cat)	UP000011712	9685	C:97%[S:67%,D:30%],F:1.4%,M:1.6%,n:6253	19645
Ailuropoda melanoleuca (Giant panda)	UP000008912	9646	C:97.4%[S:91.7%,D:5.8%],F:2%,M:0.6%,n:6253	19332
Pteropus alecto (Black flying fox)	UP000010552	9402	C:84.2%[S:83.7%,D:0.5%],F:9%,M:6.8%,n:6253	19520
Erinaceus europaeus (Western European hedgehog)	UP000079721	9365	C:95.2%[S:66.7%,D:28.4%],F:3.6%,M:1.2%,n:6253	19242
Equus caballus (Horse)	UP000002281	9796	C:97.8%[S:57.5%,D:40.3%],F:1.3%,M:0.9%,n:6253	20845
Bos taurus (Bovine)	UP000009136	9913	C:98.2%[S:34.4%,D:63.8%],F:1.2%,M:0.6%,n:6253	23844
Mustela putorius furo (European domestic ferret)	UP000000715	9669	C:96.2%[S:94.9%,D:1.3%],F:2.4%,M:1.4%,n:6253	19902
Lipotes vexillifer (Yangtze river dolphin)	UP000265300	118797	C:98.5%[S:74.2%,D:24.3%],F:1.1%,M:0.3%,n:6253	18846
Leptonychotes weddellii (Weddell seal)	UP000245341	9713	C:85%[S:50.5%,D:34.5%],F:14%,M:1%,n:6253	13162
Ursus maritimus (Polar bear)	UP000261680	29073	C:97.1%[S:71.8%,D:25.3%],F:2.6%,M:0.2%,n:6253	19368
Delphinapterus leucas (Beluga whale)	UP000248483	9749	C:98.6%[S:44%,D:54.6%],F:1.1%,M:0.4%,n:6253	17043
Odobenus rosmarus divergens (Pacific walrus)	UP000245340	9708	C:99%[S:64%,D:35%],F:0.9%,M:0.1%,n:6253	19331

Physeter macrocephalus (Sperm whale)	UP000248484	9755	C:86.3%[S:53.7%,D:32.6%],F:0.8%,M:12.9%,n:6253	20100
Tursiops truncatus (Atlantic bottle-nosed dolphin)	UP000245320	9739	C:84.1%[S:42.6%,D:41.4%],F:8.4%,M:7.6%,n:6253	17075
Loxodonta africana (African elephant)	UP000007646	9785	C:97.4%[S:73.3%,D:24.1%],F:1.9%,M:0.6%,n:4104	20015
Trichechus manatus latirostris (Florida manatee)	UP000248480	127582	C:98.1%[S:55.4%,D:42.7%],F:1.6%,M:0.3%,n:4104	19079
Ornithorhynchus anatinus (Duckbill platypus)	UP000002279	9258	C:75.7%[S:68%,D:7.7%],F:18.8%,M:5.5%,n:4104	21677
Meleagris gallopavo (Wild turkey)	UP000001645	9103	C:91.1%[S:81.4%,D:9.7%],F:5.4%,M:3.5%,n:4915	14164
Taeniopygia guttata (Zebra finch)	UP000007754	59729	C:95.4%[S:91.2%,D:4.3%],F:3.6%,M:0.9%,n:4915	17428
Anas platyrhynchos (Mallard)	UP000296049	8839	C:79.4%[S:78.5%,D:0.9%],F:7.8%,M:12.8%,n:4915	16574
Dryobates pubescens (Downy woodpecker)	UP000053875	118200	C:94.6%[S:93.7%,D:1%],F:2.2%,M:3.2%,n:4915	13097
Tinamus guttatus (White-throated tinamou)	UP000053641	94827	C:89.8%[S:88.5%,D:1.3%],F:5.5%,M:4.7%,n:4915	13377
Amazona aestiva (Blue-fronted Amazon parrot)	UP000051836	12930	C:86.6%[S:85.4%,D:1.2%],F:7.5%,M:5.9%,n:4915	16092
Calypte anna (Anna's hummingbird)	UP000054308	9244	C:95.4%[S:94.5%,D:0.9%],F:1.7%,M:2.9%,n:4915	13267
Columba livia (Rock dove)	UP000053872	8932	C:93%[S:78.5%,D:14.4%],F:5%,M:2.1%,n:4915	14619
Callipepla squamata (Scaled quail)	UP000198323	9009	C:77.6%[S:75.8%,D:1.8%],F:14.5%,M:7.9%,n:4915	16973
Aptenodytes forsteri (Emperor penguin)	UP000053286	9233	C:97.9%[S:97%,D:0.9%],F:0.8%,M:1.3%,n:4915	13704
Opisthocomus hoazin (Hoatzin)	UP000053605	30419	C:95.3%[S:94.7%,D:0.6%],F:2.1%,M:2.6%,n:4915	12773
Egretta garzetta (Little egret)	UP000053119	188379	C:96.7%[S:96%,D:0.8%],F:1%,M:2.3%,n:4915	13489
Alligator mississippiensis (American alligator)	UP000050525	8496	C:88.6%[S:67.4%,D:21.2%],F:6.8%,M:4.6%,n:3950	24656
Alligator sinensis (Chinese alligator)	UP000189705	38654	C:74.4%[S:55.1%,D:19.3%],F:2.4%,M:23.2%,n:3950	19111
Anolis carolinensis (Green anole)	UP000001646	28377	C:92.1%[S:88.4%,D:3.7%],F:5.3%,M:2.6%,n:3950	18525
Pelodiscus sinensis (Chinese softshell turtle)	UP000007267	13735	C:93.5%[S:79.1%,D:14.4%],F:4.9%,M:1.6%,n:3950	18109
Salmo salar (Atlantic salmon)	UP000087266	8030	C:97.9%[S:23.9%,D:74%],F:1.5%,M:0.6%,n:4584	47717
Oncorhynchus mykiss (Rainbow trout)	UP000193380	8022	C:77.1%[S:46.4%,D:30.7%],F:10.9%,M:12%,n:4584	46447
Gasterosteus aculeatus (Three-spined stickleback)	UP000007635	69293	C:97.5%[S:74.5%,D:23%],F:1.9%,M:0.6%,n:4584	20665
Seriola dumerili (Greater amberjack)	UP000261420	41447	C:97.6%[S:69.5%,D:28.1%],F:1.5%,M:0.8%,n:4584	23238
Takifugu rubripes (Japanese pufferfish)	UP000005226	31033	C:95.1%[S:65.4%,D:29.7%],F:2.7%,M:2.2%,n:4584	20591
Xenopus laevis (African clawed frog)	UP000186698	8355	C:95.6%[S:41.5%,D:54.1%],F:1.6%,M:2.8%,n:3950	43235
Xenopus tropicalis (Western clawed frog)	UP000008143	8364	C:62.9%[S:37.4%,D:25.4%],F:2.4%,M:34.8%,n:3950	35973
Daphnia pulex (Water flea)	UP000000305	6669	C:96.1%[S:93.8%,D:2.3%],F:1.4%,M:2.5%,n:1066	30118
Tribolium castaneum (Red flour beetle)	UP000007266	7070	C:98.6%[S:91.5%,D:7.1%],F:1.2%,M:0.2%,n:1658	16568
Bombyx mori (Silk moth)	UP000005204	7091	C:89.9%[S:89.4%,D:0.5%],F:6.8%,M:3.3%,n:1658	14773
Anopheles darlingi (Mosquito)	UP000000673	43151	C:89.4%[S:89%,D:0.4%],F:2.6%,M:7.9%,n:2799	10447
Drosophila melanogaster (Fruit fly)	UP000000803	7227	C:99.3%[S:38.2%,D:61.1%],F:0.4%,M:0.3%,n:2799	13790
Harpegnathos saltator (Jerdon's jumping ant)	UP000008237	610380	C:90.3%[S:90%,D:0.3%],F:6.4%,M:3.3%,n:4415	15029
Ooceraea biroi (Clonal raider ant)	UP000053097	2015173	C:95.9%[S:95.4%,D:0.4%],F:3.4%,M:0.8%,n:4415	16497
Papilio xuthus (Asian swallowtail butterfly)	UP000053268	66420	C:96.3%[S:94.9%,D:1.4%],F:2.4%,M:1.3%,n:1658	15265
Zootermopsis nevadensis (Dampwood termite)	UP000027135	136037	C:93.9%[S:92.8%,D:1.1%],F:1.6%,M:4.5%,n:1658	14539
Operophtera brumata (winter moth)	UP000037510	104452	C:75.9%[S:73.4%,D:2.5%],F:15%,M:9.2%,n:1658	16814
Lucilia cuprina (Green bottle fly)	UP000037069	7375	C:92.5%[S:91.5%,D:1%],F:1.8%,M:5.6%,n:2799	14353
Saccharomyces cerevisiae (strain ATCC 204508 / S288c) (Baker's yeast)	UP000002311	559292	C:98.9%[S:98.3%,D:0.6%],F:1.1%,M:0%,n:1711	6049
Emericella nidulans (strain FGSC A4 / ATCC 38163 / CBS 112.46 / NRRL 194 / M139) (Aspergillus nidulans)	UP000000560	227321	C:93%[S:92.9%,D:0.1%],F:4.5%,M:2.5%,n:4046	10557
Neurospora crassa (strain ATCC 24698 / 74-OR23-1A / CBS 708.71 / DSM 1257 / FGSC 987)	UP000001805	367110	C:99%[S:88.6%,D:10.4%],F:0.8%,M:0.2%,n:3725	9759
Yarrowia lipolytica (strain CLIB 122 / E 150) (Yeast) (Candida lipolytica)	UP000001300	284591	C:87.9%[S:87.3%,D:0.6%],F:9.8%,M:2.3%,n:1711	6449
Arachis hypogaea (Peanut)	UP000289738	3818	C:93.7%[S:17.2%,D:76.5%],F:1.7%,M:4.7%,n:1440	71122

Musa acuminata subsp. Malaccensis (Wild banana)	UP000012960	214687	C:86.7%[S:76.5%,D:10.3%],F:4.7%,M:8.5%,n:1440	36474
Arabidopsis thaliana (Mouse-ear cress)	UP000006548	3702	C:99.6%[S:59.1%,D:40.5%],F:0.2%,M:0.2%,n:1440	27466
Oryza sativa subsp. indica (Rice)	UP000007015	39946	C:94.8%[S:93.6%,D:1.2%],F:2.4%,M:2.8%,n:1440	37344
Zea mays (Maize)	UP000007305	4577	C:96.4%[S:49.4%,D:46.9%],F:2.1%,M:1.5%,n:1440	39400
Triticum aestivum (Wheat)	UP000019116	4565	C:99.4%[S:1.4%,D:98%],F:0.1%,M:0.5%,n:1440	105061
Physcomitrella patens subsp. patens (Moss)	UP000006727	3218	C:67.8%[S:52%,D:15.8%],F:3.3%,M:28.9%,n:1440	30857
Emiliania huxleyi (Pontosphaera huxleyi)	UP000013827	2903	C:74.9%[S:0.7%,D:74.3%],F:14.2%,M:10.9%,n:303	35676
Dictyostelium discoideum (Slime mold)	UP000002195	44689	C:96%[S:92.4%,D:3.6%],F:0.7%,M:3.3%,n:303	12739
Chlamydomonas reinhardtii (Chlamydomonas smithii)	UP000006906	3055	C:96%[S:90.1%,D:5.9%],F:2.3%,M:1.7%,n:303	17614
Thalassiosira pseudonana (Marine diatom)	UP000001449	35128	C:34.2%[S:33.3%,D:0.9%],F:1.3%,M:64.5%,n:234	11717
Plasmodium falciparum (isolate 3D7)	UP000001450	36329	C:25.6%[S:25.2%,D:0.4%],F:0.4%,M:73.9%,n:234	5376
